# Longitudinal assessment of network reorganizations and language recovery in postoperative patients with glioma

**DOI:** 10.1093/braincomms/fcac046

**Published:** 2022-04-06

**Authors:** Binke Yuan, Nan Zhang, Fangyuan Gong, Xindi Wang, Jing Yan, Junfeng Lu, Jinsong Wu

**Affiliations:** 1 Key Laboratory of Brain, Cognition and Education Sciences, Ministry of Education, Guangzhou, China; 2 Institute for Brain Research and Rehabilitation, South China Normal University, Guangzhou, China; 3 Department of Neurosurgery, The First Affiliated Hospital of USTC, Division of Life Sciences and Medicine, University of Science and Technology of China, Hefei, Anhui, China; 4 Glioma Surgery Division, Neurologic Surgery Department, Huashan Hospital, Shanghai Medical College, Fudan University, Shanghai, China; 5 National Key Laboratory of Cognitive Neuroscience and Learning, Beijing Normal University, Beijing, China; 6 Beijing Key Laboratory of Brain Imaging and Connectomics, Beijing Normal University, Beijing, China; 7 IDG/McGovern Institute for Brain Research, Beijing Normal University, Beijing, China; 8 Department of MRI, The First Affiliated Hospital of Zhengzhou University, Zhengzhou, China; 9 Brain Function Laboratory, Neurosurgical Institute of Fudan University, Shanghai, China; 10 Shanghai Key Laboratory of Brain Function Restoration and Neural Regeneration, Shanghai, China

**Keywords:** glioma, language recovery, resting-state fMRI, functional network

## Abstract

For patients with glioma located in or adjacent to the linguistic eloquent cortex, awake surgery with an emphasis on the preservation of language function is preferred. However, the brain network basis of postoperative linguistic functional outcomes remains largely unknown. In this work, 34 patients with left cerebral gliomas who underwent awake surgery were assessed for language function and resting-state network properties before and after surgery. We found that there were 28 patients whose language function returned to at least 80% of the baseline scores within 3 months after surgery or to 85% within 6 months after surgery. For these patients, the spontaneous recovery of language function synchronized with changes within the language and cognitive control networks, but not with other networks. Specifically, compared with baseline values, language functions and global network properties were the worst within 1 month after surgery and gradually recovered within 6 months after surgery. The recovery of connections was tumour location dependent and was attributed to both ipsihemispheric and interhemispheric connections. In contrast, for six patients whose language function did not recover well, severe network disruptions were observed before surgery and persisted into the chronic phase. This study suggests the synchronization of functional network normalization and spontaneous language recovery in postoperative patients with glioma.

## Teaser

For patients with glioma involving language networks, Yuan *et al*. investigated postoperative brain network bases for diverse time courses of spontaneous recovery of language functions. The spontaneous language recovery individually and network-specifically synchronized with the changes of language and cognitive control networks.

## Graphical Abstract

**Graphical Abstract:**
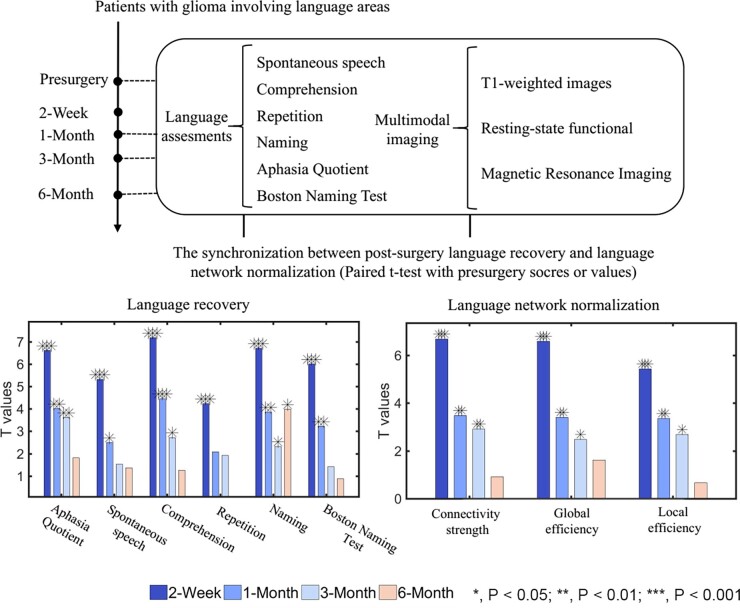

## Introduction

A brain tumour is a mass or growth of abnormal cells in the brain. Gliomas are the most common intra-axial tumours.^1^ Diffusive and progressive glioma infiltration of ‘eloquent’ brain areas leads to behavioural or cognitive deficits.^2,3^ The preservation of basic functions (e.g. language and sensorimotor functions) is critical to neurosurgical procedures. Awake surgery is now the first therapeutic option for patients with glioma, with the aims being maximum resection and minimum functional injury, as well as prolonging survival time and improving the of postoperative life.^4–9^

Functional outcomes after awake surgery have been explored in several studies and the recovery of neurological/neuropsychological functions has been shown to differ depending on functional domains.^5,10–12^ For glioma involving language areas, transient language deficits are generally observed in the (sub) acute phase and typically recover to a level equal to or similar to the preoperative level (baseline scores) within 3 months after surgery.^5,6,8,10,13–15^ A small portion of patients will suffer a permanent severe language deficit in the chronic phase. Factors related to postoperative language recovery include preoperative status and baseline deficits, tumour grade, tumour variables (size, location, histology oedema), neurosurgical procedures, postoperative chemo and/or radiation treatment and recovery time.^5,6,8,10,11,13,16^ However, the neural substrates underlying different language recovery processes remain largely unknown.^12,17^

Longitudinal neuroimaging studies of language-related changes in brain activity after surgery are still relatively scarce. Task-based functional MRI (fMRI) is now the most widely applied non-invasive technique for assessing pre- and postsurgical functional mapping of language sites.^12,18^ Preoperative gliomas in language areas have been shown to induce different patterns of functional reshaping,^12,13,19,20^ including the recruitment of perilesional regions as local reorganization, changes in activation patterns of the contralesional homologous regions^21,22^ and remote recruitment of other remote brain regions such as the cerebellum.^2^ However, pattern reshaping after surgery remains to be addressed. Deverdun *et al.*^23^ showed that among 32 patients with low-grade gliomas (LGGs) whose language performance recovered to a level equal or similar to the preoperative level within 3 months, 81.2% did not show any differences in pre/post-surgery (3 months) activation during a picture-naming task. The remaining patients showed differences in activation at several locations, but with no unique plasticity pattern. A recent study by Voets *et al.*^24^ showed that among 19 patients with glioma involving language networks, patient-unique language activation patterns were observed after surgery. These findings demonstrated that a systematic pattern of plasticity associated with tumour growth or resection might not exist, especially in patients with LGG whose functional reshaping is sufficient. Rather, each brain reorganizes itself differently depending on the individual and lesion-related factors.^13,17,21,25,26^ Nevertheless, many important issues remain to be addressed. First, little work investigated the network reorganizations in the (sub) acute phase. Considering that most patients with glioma typically recover within 3 months after an awake surgery, the cognitive status and the network patterns in the (sub) acute phase may be more predictive of longitudinal outcomes. Second, tumour grade is a leading predictor of neuroplasticity and cognitive outcomes.^2,27,28^ According to the World Health Organization (WHO) grading system, Grades I and II brain tumours are often referred to as LGG, while Grades III and IV are described as high-grade gliomas (HGGs). Studies are needed to elucidate whether brain recovery differs depending on whether the tumour grade is high or low.^5^ Third, modern network science shows that the brain is organized into hierarchical, integrated and interconnected large-scale networks.^29,30^ Specifically, language processing is supported by left lateralized, delocalized but interconnected cortico–subcortical regions and bilaterally distributed connections.^9,26,31–34^ Large-scale network disruptions in neural substrates are believed to underlie behavioural and cognitive deficits after brain damage.^19,20,35,36^ Until now, the details of postsurgical network reorganization during language recovery remained unknown in patients with glioma.

Motivated by the shift in language processing theories from a localizationist perspective to a network-centric perspective, the current study was designed to investigate the functional network basis of postoperative language recovery in patients with glioma using resting-state functional connectivity analyses. To this end, language and rs-fMRI data were acquired from a sample of 34 patients with left cerebral glioma before surgery and 2 weeks, 1 month, 3 months and 6 months after surgery. All patients underwent awake surgery and language mapping via intraoperative stimulation. We studied the spontaneous recovery of language function and changes within functional networks. We analysed two networks of interest: a functionally specialized language network and a domain-general cognitive control network, which has been shown to play an important role in language production and comprehension.^26,37–40^ We hypothesized that the extent of language deficits and the recovery would be coupled with changes within the language network and its supporting system (i.e. cognitive control network).

## Materials and methods

### Participants

Patients and controls were enrolled from the Huashan hospital. All procedures strictly followed the Declaration of Helsinki. This study was approved and supervised by the Huashan Institutional Review Board (2017-423). Written informed consent was obtained from all participants or their legal guardians.

Patients were prospectively screened for the following inclusion criteria: (i) pathologically confirmed glioma (based on the 2016 WHO Classification of tumours of the CNS) in the left cerebral hemisphere^41^; (ii) no history of chemotherapy or radiation treatment before surgery; (iii) age between 18 and 75 years; (iv) right-handedness confirmed by the Edinburgh Handedness Inventory; (v) no symptoms of motor impairment, as indicated by a Grade V on the Medical Research Council Scale for Muscle Strength; (vi) Chinese Han nationality; (viii) no history of brain surgery; (viii) no midline shifts observed in structural images, as confirmed by the *in situ* location of midline structures of the brain (corpus callosum, septa pellucidum, third ventricle, hypothalamus and pineal region); (ix) both structural and functional images that covered the whole brain, especially the whole cerebellum; (x) good cooperation during the linguistic/cognitive evaluations; (xi) no history of other major neurological or psychiatric disorders and (xii) no history of alcohol or drug abuse.

Thirty-four patients met the inclusion criteria, including 20 patients with Grade II gliomas, 7 patients with Grade III gliomas and 7 patients with Grade IV gliomas. All patients underwent awake craniotomies with intraoperative stimulation for language mapping. The detailed surgical procedures have been documented in our previous studies.^7,8^

**Figure 1:**
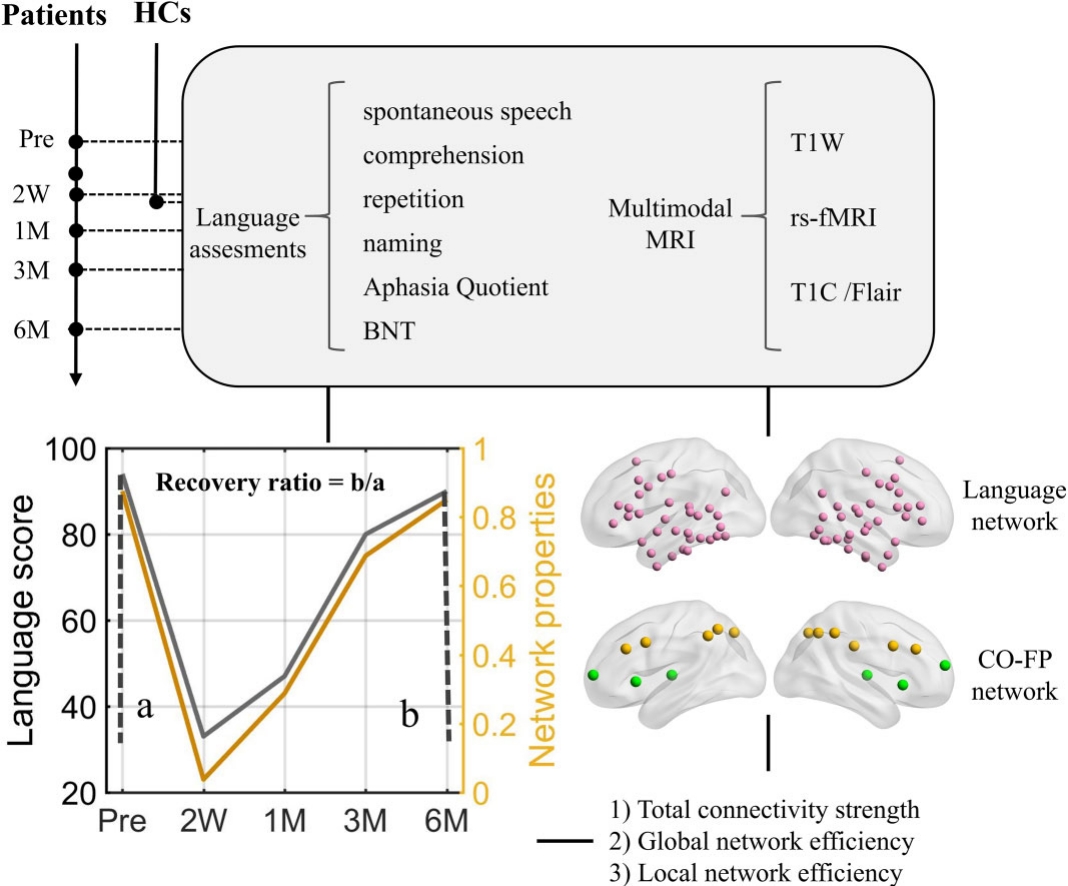
**The timeline of data acquisitions and brain network-language synchronization analyses.** Language functions were assessed using the BNT and the Aphasia Battery for Chinese speakers. Three global network properties, the FC strength, global and local network efficiencies were calculated for the two networks, i.e. language network and CO-FP network.^42^ Brain network-language synchronization analyses included the recovery trajectories of language scores and network properties and the association of AQ and network recovery ratios.

Patients were studied at least at three-time points (Fig. 1)—preoperatively and in the subacute phase (i.e. 2 weeks and/or 1 month after surgery) and chronic phase (i.e. 3 months and/or 6 months after surgery). At each time point, clinical and language evaluations, as well as structural and fMRI data were acquired. The demographic and clinical characteristics and language performances are summarized in Tables 1 and 2 and Supplementary material 2. Twenty-six sex- and age-matched healthy controls were recruited for a single run. There was no significant difference in sex (*P* = 0.28), age (*P* = 0.61) or education (*P* = 0.62) between patients and control subjects.

**Table 1:** Demographic, clinical characteristics and language scores of patients

	Controls (*n* = 26)	Pre (*n* = 34)	2 weeks (*n* = 19)	1 month (*n* = 19)	3 months (*n* = 26)	6 months (*n* = 9)
Age	42 ± 12.41	43.64 ± 12.28	44.16 ± 12.6	42.81 ± 12.56	43.81 ± 11.56	41.67 ± 14.95
Gender (M/F)	14/12	23/11	15/4	11/8	18/8	6/3
Education	11.31 ± 3.53	11.91 ± 5.37	12.74 ± 5.58	11.89 ± 5.07	11.69 ± 5.49	12.56 ± 4.9
Tumour grade	—	II/III/IV = 20/7/7	II/III/IV = 12/3/4	II/III/IV = 11/4/4	II/III/IV = 15/4/7	II/III/IV =6/3/0
Tumour (lesion) volume (mm^3^)	—	70.88 ± 53.34	85.07 ± 34.72	70.46 ± 44.87	32.82 ± 19.14	56.21 ± 50.28
Tumour location (F/T/P/Ins)	—	14/10/5/5				
Tumour resection (GTR/STR/PR)	—	II = 11/2/6				
	—	III = 6/1/0				
	—	IV = 6/0/1				
KPS	100 ± 0	96.5 ± 4.81	82.63 ± 20.23	94.74 ± 11.72	97.14 ± 4.6	97.14 ± 4.88
MMSE	28.46 ± 1.61	26.91 ± 9.59	12 ± 8.03	21.53 ± 6.75	24.39 ± 6.2	19.43 ± 8.83
BNT	26.89 ± 2.78	19.29 ± 6.16	9 ± 8.41	14.42 ± 7.43	17.93 ± 7.7	14.57 ± 8.38
AQ	98.54 ± 2.09	90.1 ± 9	54.4 ± 22.73	75.4 ± 16.05	85.39 ± 12.47	77.42 ± 14.57
SS	19.73 ± 0.72	17.32 ± 2.46	10.74 ± 4.95	14.89 ± 4.5	16.46 ± 3.12	15.2 ± 3.09
Comprehension	226.42 ± 5.41	210.44 ± 23.08	139.74 ± 43.46	186.53 ± 27.2	203.18 ± 28.37	198.43 ± 33.84
Repetition	99.73 ± 0.72	95.38 ± 7.25	60.26 ± 33.55	83.05 ± 21.98	89.11 ± 17.21	86 ± 20
Naming	96.77 ± 3.50	87.96 ± 11.03	41.16 ± 27.5	71.63 ± 17.78	81.64 ± 15.36	79.43 ± 16.87

M, male; F, female or prefrontal; T, temporal; P, parietal; Ins, insular; GTR, gross total resection; STR, sub-total resection; PR, partial resection; KPS, Karnofsky performance scale; BNT, Boston Naming Test; MMSE, mini-mental state examination; AQ, aphasia quotient.

**Table 2:** Individual demographic and clinical characteristics of patients

	ID	Sex	Age	Education	WHO grade	Location	Tumour resection	AQ				
								Pre	2 weeks	1 month	3 months	6 months
Good recovery	P009	M	30	12	2	Ins	PR	84.91	—	79.91	79.18	84.05
	P011	M	23	9	2	F	GTR	90.88	—	72.09	—	91.33
	P016	M	36	12	2	F	STR	95.78	87.48	—	—	93.04
	P017	M	38	14	3	F	GTR	97.2	42.74	—	85.1	—
	P018	F	67	3	3	T	GTR	82.16	51.26	—	—	70.81
	P030	M	46	6	4	F	GTR	82.07	—	65.83	73.71	—
	P043	M	43	18	2	F	GTR	90.4	68.55	—	91.82	—
	P046	F	32	9	2	F	GTR	95.1	—	88.42	92.7	—
	P049	M	29	16	3	F	GTR	97.2	—	85.9	97.77	—
	P051	M	52	16	4	T	GTR	80.52	59.28	—	79.82	—
	P053	F	29	14	2	T	GTR	100	81.58	—	100	—
	P071	M	41	16	2	Ins	STR	96.85	—	84.63	95.2	—
	P072	M	59	1	2	T	PR	68.54	39.02	—	56.6	—
	P075	M	36	19	2	Ins	PR	98.77	14.83	—	98.03	—
	P081	M	61	6	4	F	PR	87.95	55.25	—	87.36	—
	P094	M	50	16	3	T	GTR	98.2	67.37	—	92.21	—
	P096	F	39	9	2	P	STR	87.93	24.95	—	77.79	—
	P100	M	33	21	2	T	GTR	97.2	81.9	—	97.8	—
	P103	F	39	4	3	F	GTR	84.36	—	41.61	71.46	—
	P105	M	28	12	2	F	GTR	97.1	64.57	—	94.42	—
	P201	M	55	12	2	Ins	PR	94.81	—	84.51	90.13	—
	P204	F	59	12	4	P	GTR	91.82	—	87.14	86.17	—
	P210	F	40	9	2	T	GTR	95.4	—	87.5	87.08	—
	P217	F	31	16	2	T	GTR	96.53	79.62	97.76	99.45	—
	P218	M	28	19	4	F	GTR	93.06	62.77	84.31	92.52	—
	P219	M	42	16	2	Ins	PR	99.05	14.37	91.96	96.29	—
	P220	M	50	9	2	F	PR	95.97	—	75.27	89.42	—
	P221	M	62	12	2	F	GTR	79.89	23.39	81.07	84.25	—
Poor recovery	P028	F	36	16	2	P	GTR	97.42	—	63.52	—	70.58
	P044	M	49	12	2	P	GTR	95.4	49.85	—	80.98	—
	P047	F	60	0	3	T	STR	66.89	—	50.23	52.39	—
	P048	F	63	14	3	F	GTR	70.37	—	45.87	—	51.17
	P101	M	42	19	4	P	GTR	90.67	—	65.07	72.25	—
	P106	M	56	6	4	T	GTR	84.43	64.9	—	69.98	—

F, prefrontal; T, temporal; P, parietal; Ins, insula; GTR, gross total resection; PR, partial resection; STR, sub-total resection.

### Language assessments

Language function was assessed in detail using the Boston Naming Test (BNT) and the Aphasia Battery of Chinese (ABC) speakers. The BNT (range, 0–30) is one of the most widely used standardized aphasia measures in clinical practice, particularly for naming ability.^43^ The ABC is the Chinese standardized adaptation of the Western Aphasia Battery^2,8^ and includes subscores for spontaneous speech (*S*_SS_) (range, 0–20), comprehension (*S*_Com_) (range, 0–230), repetition (*S*_Rep_) (range, 0–100) and naming (*S*_Nam_) (range, 0–100). The aphasia quotient (AQ) (range, 0–100) can be calculated from these items to reflect the global severity: AQ = (S_ss_ + S_com_/23 + S_rep_/10 + S_nam_/10) * 2. Participant demographics and language performance are summarized in Table 2.

We grouped patients into subgroups depending on the recovery process during the chronic phase: (i) good recovery—patient AQs returned to 80% of his or her baseline (i.e., preoperative phase) scores by 3 months after surgery or to 85% by 6 months^44–47^; (ii) poor recovery—patient AQs did not return to 80% of baseline scores by 3 months after surgery or to 85% by 6 months.

### Image acquisition

All neuroimaging data were obtained using a Siemens Magnetom Verio 3.0 T MRI scanner (Siemens Medical Solutions, Erlangen, Germany). For patients with LGGs, high resolution T_1_-weighted and T_2_-weighted fluid-attenuated inversion recovery (T_2_-weighted FLAIR) images were acquired with the following parameters. T_1_-weighted images: axial magnetization-prepared rapid gradient-echo sequence; repetition time (TR) = 1900 ms; echo time (TE) = 2.93 ms; flip angle (FA) = 9°; inversion time (TI) = 900 ms; field of view (FOV) = 250 × 219 mm; matrix size = 256 × 215; slice thickness = 1 mm; voxel size = 1 × 1 × 1 mm; slice number = 176 and scanning time = 7 min 47 s. T_2_-weighted FLAIR images: TR = 9000 ms; TE = 99 ms; FA = 150°; TI = 2500 ms; FOV = 240 × 214 mm; matrix size = 256 × 160; slice thickness = 2 mm; voxel size = 0.9 × 1.3 × 2.0 mm; slice number = 66 and scanning time = 7 min 30 s. For the HGG group, T_1_-weighted sequence images with contrast (gadopentetate dimeglumine) were acquired with the same parameters.

For all participants, resting-state fMRI (rs-fMRI) images were acquired using the following parameters: TR = 2000 ms; TE = 35 ms; FA = 90°; FOV = 240 × 240 mm; matrix size = 64 × 64; thickness/gap = 4/1 mm; voxel size = 3.3 × 3.3 × 5.0 mm; slice number = 33; scanning time = 8 min and number of time points = 240. All participants were required to remain still with their eyes closed and stay awake.

### Structural data processing

For each patient, the tumour territory was manually drawn (by neurosurgeons N.Z. and F.G.) slice by slice on the native 3D T_1_-weighted images. Manual tumour drawing was performed based on the contrast-enhancing tumour areas or the FLAIR hyperintense areas (necrotic areas but not peritumoral oedema were included). For 3D T_1_-weighted images without glioma enhancement, T_2_-weighted FLAIR images were first coregistered to the 3D T_1_-weighted images to serve as a visual reference. This procedure was performed manually using RANO criteria as a reference.^48^ After manually tracing the tumour, we created a 3D T_1_-weighted volume that lacked the tumour area (set to 0). Each 3D T_1_-weighted volume without a tumour area was segmented into grey matter (GM), white matter (WM), CSF, bone, soft tissue and air/background using SPM12 (https://www.fil.ion.ucl.ac.uk/spm). We then used Diffeomorphic Anatomical Registration using Exponentiated Lie algebra (DARTEL) for registration, normalization and modulation.^49^ DARTEL has been shown to provide a more accurate normalization than Unified Segmentation with cost function masking in patients with focal lesions.^50^ A customized template was generated using the average tissue probability maps across all participants, and then each participant’s segmented map was warped into the template. This procedure was repeated until we generated the best study-specific template. The images were then modulated according to the Jacobian determinants to ensure the conservation of regional differences in the absolute amounts of GM. Finally, the registered images were transformed to the Montreal Neurological Institute (MNI) space. The native tumour mask was spatially normalized to the standard MNI space by applying the deformation field estimated by segmentation.

### Functional data processing

Only fMRI signals in intact voxels were considered in the following analyses. The first 10 volumes were discarded, and then slice timing and motion correction were performed. The motion-corrected functional images were coregistered to the 3D T_1_-weighted and then spatially normalized into the MNI space by applying the deformation field as estimated by segmentation. The normalized images were spatially smoothed using a Gaussian kernel (full-width-at-half-maximum = 6 mm). The linear trend was removed, and the nuisance signals [36 parameters, including the *x*, *y*, *z* translations, and rotations + WM/CSF/global time courses (nine parameters), plus their temporal derivatives (nine parameters) and the quadratic terms of 18 parameters] were removed by linear regression from each voxel’s time course^51,52^ (https://github.com/sandywang/RegressOutForRfMRIwithTumor). Then, temporal band-pass filtering (0.01–0.1 Hz) was performed on the residuals. A ‘scrubbing’ procedure was additionally adopted to reduce any head motion artefacts.^53,54^ Specifically, rs-fMRI volumes showing sudden head motion were discarded along with one volume before and two volumes after the bad volume. Sudden head motion was defined as a frame-wise displacement > 0.5 mm.^53,55,56^ After removing bad volumes, no patient had fewer than 140 remaining volumes.

### Networks of interest and functional connectivity analysis

#### Language network

The putative language network was defined based on the meta-analysis reported by Fan *et al*.^57^ In a whole-brain connectivity-based parcellation (i.e. the Brainnetome Atlas, covering the cortical and subcortical regions, no cerebellar parcels), for each parcel, Fan *et al.* performed meta-analyses based on behavioural domain and paradigm-class meta-data labels from the BrainMap database. We extracted all parcels that exhibited significant activation in language-related behavioural domains (i.e. language or speech) or paradigm classes (e.g. semantic, word generation, reading or comprehension). Most of these cortical parcels are in the left hemisphere and their extents are highly similar to those identified by other researchers based on other meta-results.^58–61^ Considering the recruitment of right hemisphere language regions for compensation and bilateral processing, parcels of the left-lateralized language region homologues (i.e. parcels in the right frontal and temporal lobes) were also included.^22,25^ Altogether, a symmetric network, including 68 cortical regions of interest (ROIs) covering the bilateral inferior frontal gyrus (IFG), middle frontal gyrus (MFG), superior frontal gyrus (SFG), superior temporal gyrus (STG), middle temporal gyrus (MTG), inferior temporal gyrus (ITG), precentral gyrus (PrG),^9,62^ postcentral gyrus (PoG) and inferior parietal lobule (IPL) were defined (Supplementary Table 1 and Fig. 1).

#### CO-FP network

The locations of the cingulo-opercular (CO) and fronto-parietal (FP) networks were from Dosenbach *et al*.,^42^ which identified several regions active during different stages of cognitive control tasks. The CO network included seven ROIs collectively located in the dorsal anterior cingulate, bilateral anterior insula, bilateral anterior prefrontal cortex and bilateral anterior thalamus. The FP network included 11 ROIs collectively located in the bilateral dorsolateral prefrontal cortex, bilateral IPL, bilateral intraparietal sulcus, bilateral IFG, bilateral precuneus and middle cingulate cortex. The coordinates were transformed from Talairach to MNI space (Supplementary Table 2 and Fig. 1) using icbm2tal transform.^63,64^

For each network of interest, an individual functional connectivity network was constructed by calculating Pearson’s correlation coefficients between pairwise nodes. The *r*-values in each matrix were transformed to *z*-values using Fisher’s *r*-to-*z* transformation.

### Global network properties: total connectivity strength, global and local network efficiency

A correlation threshold (*r* > 0.2) was used to eliminate weak connections that might have arisen from noise. (i) Total connectivity strength (FC strength). The FC strength of a network reflects the recovery or rewiring of functional connectivity during recovery, which was calculated by summing all functional connectivity strengths of the suprathreshold connections into one value.^65^ (ii) Global network efficiency (gE). The gE reflects the capability for parallel information transfer and functional integration.^66^ In the current study, we used a weighted network rather than a binarized network to conserve all connectivity information. The global efficiency was defined as the average of the inverses for all weighted shortest path lengths (the minimal number of edges that one node must traverse to reach another) in the thresholded matrix. (iii) Local network efficiency (lE). The lE reflects relative functional segregation and is defined as the average of the global efficiency of each node’s neighbourhood sub-graph.

To assess the disruption and recovery of each network property, we calculated the *Z*-scores against the corresponding values in the control group: , where *P* is the patient value, and *μ* and *δ* are the mean and standard deviation of the control subjects, respectively.

### Recovery ratio

To quantify the AQ and network recoveries after surgery, a recovery ratio was calculated.^67^ The AQ or network recovery ratio was calculated by dividing the amounts of total recovery by the transient declines, where total recovery was the chronic score (values) at 3 or 6 months subtracted by the subacute score (values) at 2 weeks (or 1 month) and the transient decline was the preoperative score (values) subtracted by the subacute score (values) at 2 weeks (or 1 month).

### Statistical analysis

Independent two-sample *t*-tests were used to compare age and education in patients with controls and also used to evaluate the severity of language deficits and network properties changes before and after surgery. Pearson *χ*^2^-tests were used to compare sex composition between the two groups. Paired *t*-tests with preoperative baseline scores (or values) were used to track the changes in patients’ language scores and global network properties after surgery. Partial correlations between the AQ recovery ratio and network recovery ratio, and between the amounts of language recovery and the amounts of network recovery after surgery were calculated, with sex, age, education and tumour grade as covariates.

### Validation analysis

We validated our main results by further considering the following variables: (i) network specificity. To confirm the network specificity of language recovery, we also examined changes in the default mode network (DMN) and motor execution network (MEN) as an internal reference (Supplementary Fig. 1). The regions comprising the DMN (Supplementary Table 3) were obtained from a meta-analysis of DMN connectivity,^68^ and included bilateral posterior cingulate cortex, ventromedial prefrontal cortex and temporoparietal junction. The regions comprising the MEN (Supplementary Table 4) were from Wang *et al*.^69^ and included left primary motor cortex, bilateral dorsolateral and ventrolateral premotor cortex, bilateral superior parietal lobule, bilateral basal ganglia, bilateral thalamus, bilateral anterior inferior cerebellum, PoG, bilateral dentate nucleus and superior cerebellum. (ii) Global signal removal. Global signal removal is a controversial preprocessing step and recent work suggests that different preprocessing strategies may provide complementary insights into functional brain organization.^70^ Global signal removal was performed in the main analysis to suppress the motion effect, and we also reanalysed our data without regressing out the global signal. (iii) To examine whether our main results depend on the choice of correlation threshold, we recomputed network properties using two different correlation thresholds (*r* > 0.1 and *r* > 0.3).

### Data availability

Behavioural data are publicly available in Supplementary material 2. The normalized functional and structural MRI data are available on request from the corresponding author.

## Results

### Patient characteristics, language recovery trajectories and tumour anatomy

**Figure 2:**
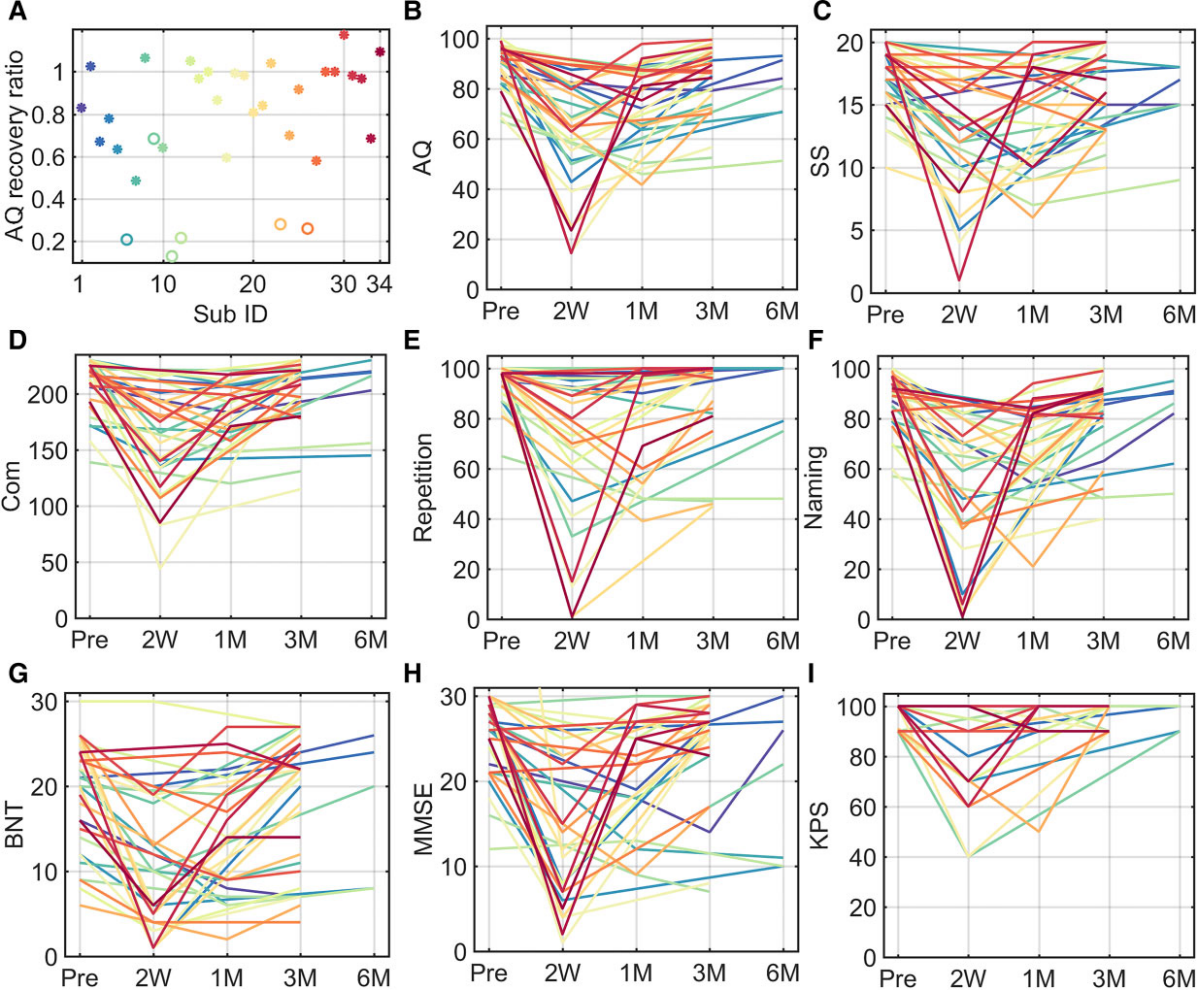
**The recovery trajectories of language, MMSE and KPS for 34 patients.** (**A**) Each dot represents an individual, with a colour corresponding to the line colour. (**B–I**) Each line represents an individual with a unique colour. AQ, aphasia quotient; SS, spontaneous speech; Com, comprehension; BNT, Boston Naming Test; KPS, Karnofsky performance scale; MMSE, mini-mental state examination.

Figure 2 shows the language recovery ratios and trajectories. Before surgery, 17 out of 34 patients had AQ scores <93.8. In the acute phase, transient declines were observed in all patients and the amount of recovery differed both among patients and the terms of language function. Twenty-eight patients exhibited good recovery and six patients did not recover well. There were two patients (P204, P210) whose language functions have well recovered within 1 month. Thus, the AQ ratios for the two patients were set to 1. The AQ recovery ratios for patients with good language recovery were generally higher (0.49–1.17) than for those whose language did not well recover (0.13–0.68).

**Figure 3:**
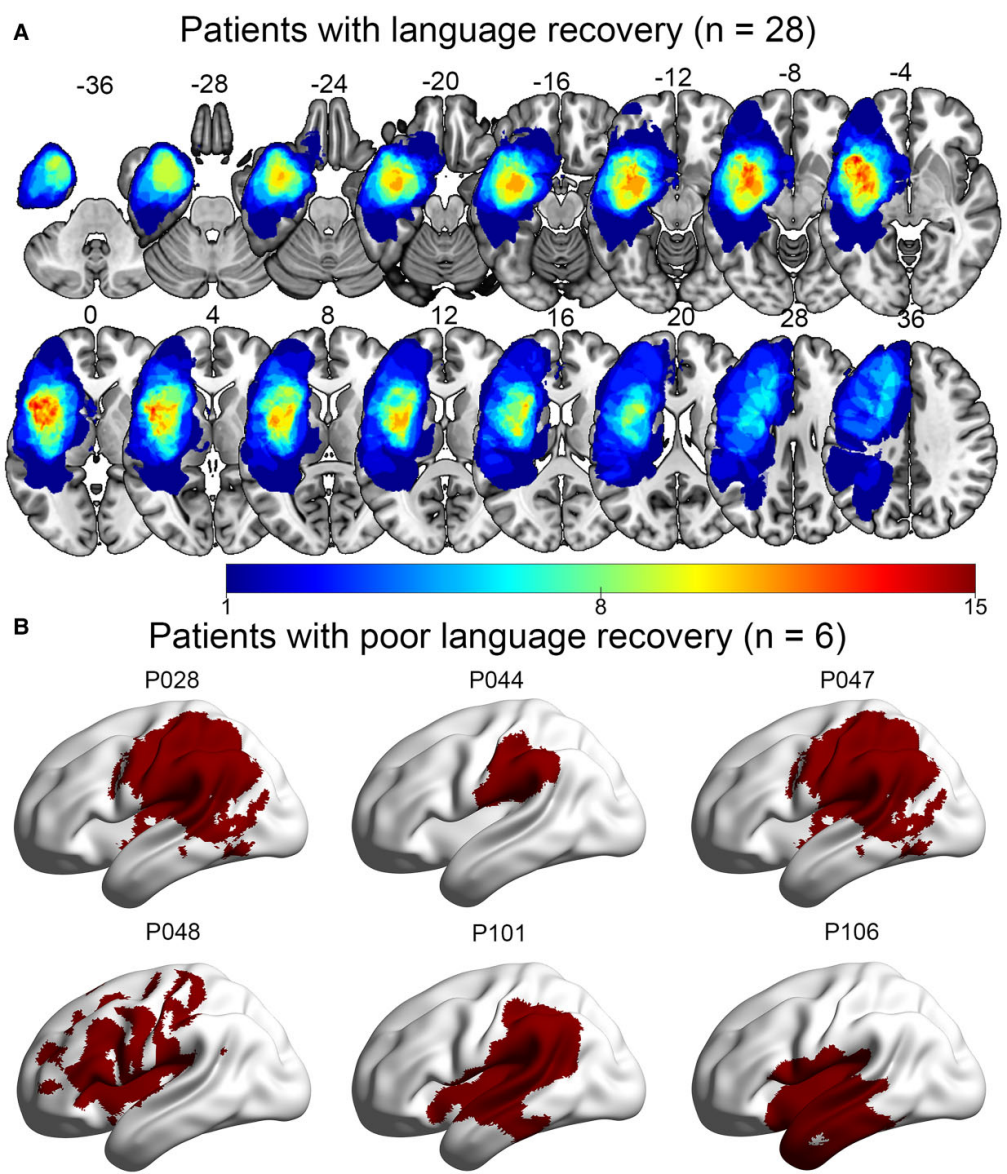
**Tumour anatomy.** (**A**) Tumour masks for patients with good language recovery. All masks in MNI space were then stacked and binarized to construct a tumour-overlapping image in which each voxel was identified as part of the tumour region from at least one patient. (**B**) Tumour masks for patients with poor language recovery.

Figure 3 shows tumour anatomy. Of the 28 patients who showed good language recovery, 18 had LGGs and 10 had HGGs. Among the 18 patients with LGGs, we identified seven frontal gliomas, five temporal gliomas, one parietal glioma and five insular gliomas. Among the 10 patients with HGGs, there were six frontal gliomas, three temporal gliomas and one parietal glioma. For the six patients who did not recover well, four had HGGs (one frontal glioma, two temporal gliomas and one parietal glioma) and two had LGGs (two parietal gliomas).

### Patients with good language recovery showed synchronized changes in global network properties within the language and CO-FP networks

**Figure 4:**
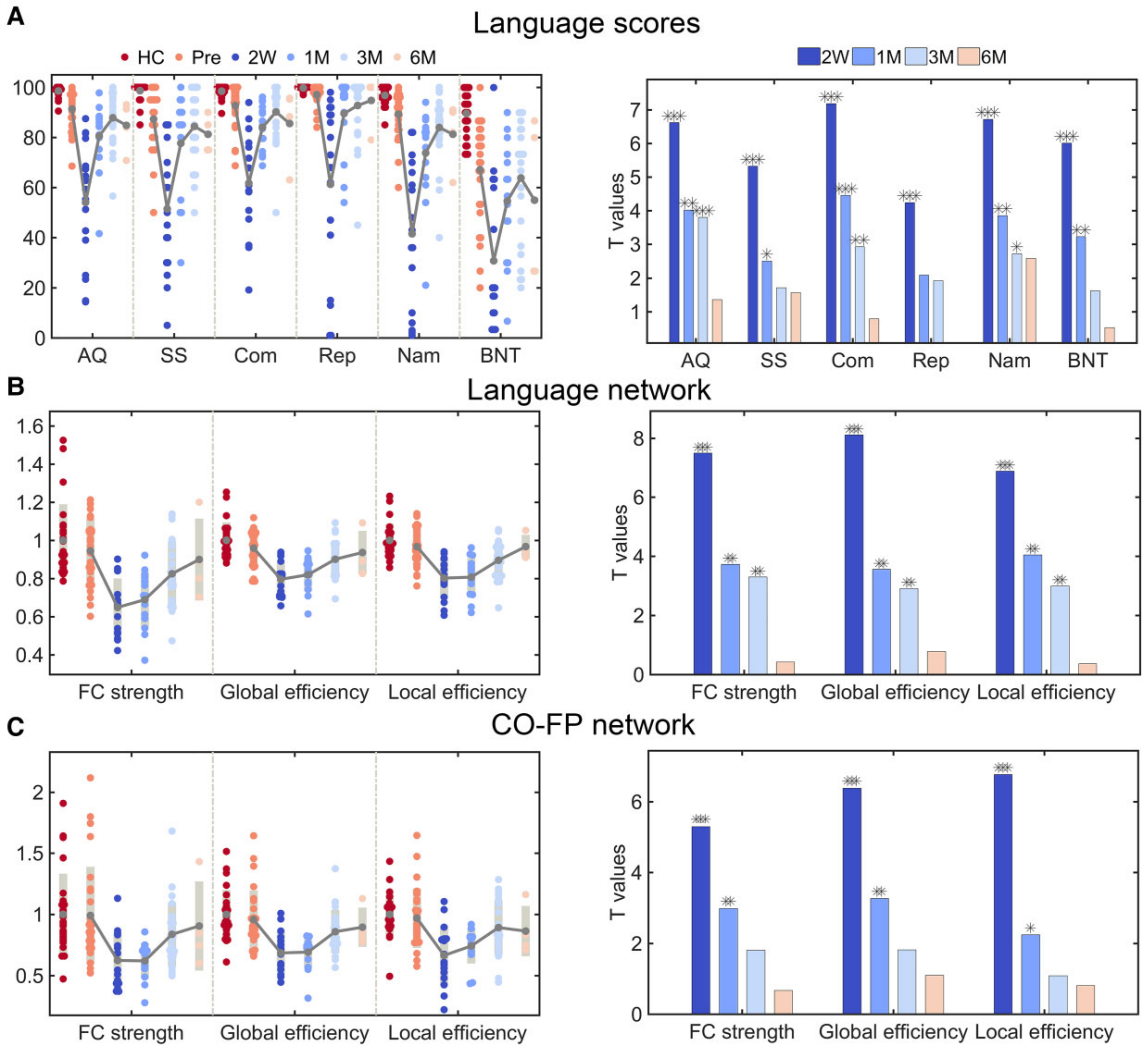
**Language scores and global network properties (*Z*-scores) for the 28 patients with a good language recovery.** (**A**) Language scores. (Left) Individual scores for the language skills and the AQ in patients and control subjects. The grey line connects the mean values. (Right) Paired *t*-values over time from the comparisons of each language function measure with preoperative baseline scores. **(B**) and (**C**) The changes of the three global network properties of language network and CO-FP network, respectively. The *t*-values were obtained by performing paired *t*-tests with the corresponding preoperative data. For illustration purposes, the language scores of SS, Com and BNT were scaled to 100. Note that the sample sizes vary in each observation, *n* = 17 in 2 weeks, *n* = 15 in 1 month, *n* = 25 in 3 months and *n* = 4 in the 6 months after surgery. AQ, aphasia quotient; SS, spontaneous speech; Com, comprehension; BNT, Boston Naming Test. *P < 0.05; **P < 0.01; ***P < 0.001.

Compared with HCs, preoperative language function was significantly lower for the 28 patients who showed good language recovery (AQ: two-sample *t*-test, *t*_52_ = 4.61, *P* < 0.001; SS: *t*_52_ = 4.54, *P* < 0.001; comprehension: *t*_52_ = 3.37, *P* = 0.002; repetition: *t*_52_ = 3.47, *P* = 0.001; naming: *t*_52_ = 3.88, *P* < 0.001; BNT: *t*_52_ = 5.21, *P* < 0.001). They reached the worst levels at 2 weeks after surgery (AQ: paired *t*-test with preoperative scores, *t*_16_ = 6.62, *P* < 0.001; SS: *t*_16_ = 5.32, *P* < 0.001; comprehension: *t*_16_ = 7.18, *P* < 0.001; repetition: *t*_16_ = 4.4, *P* < 0.001; naming: *t*_16_ = 6.72, *P* < 0.001; BNT: *t*_16_ = 6.01, *P* < 0.001), were partially recovered at 1 month (AQ: paired *t*-test with preoperative scores, *t*_14_ = 4.02, *P* = 0.001; SS: *t*_14_ = 2.51, *P* = 0.025; comprehension: *t*_14_ = 4.46, *P* < 0.001; repetition: *t*_14_ = 2.09, *P* = 0.055; naming: *t*_14_ = 3.85, *P* = 0.002; BNT: *t*_14_ = 3.23, *P* = 0.006) and well recovered at 3 months (AQ: paired *t*-test with preoperative scores, *t*_24_ = 3.80, *P* < 0.001; SS: *t*_24_ = 1.71, *P* = 0.01; comprehension: *t*_24_ = 2.93, *P* = 0.007; repetition: *t*_24_ = 1.92, *P* = 0.07; naming: *t*_24_ = 2.72, *P* = 0.01; BNT: *t*_2*4*_ = 1.62, *P* = 0.12) and at 6 months (AQ: paired *t*-test with preoperative scores, *t*_3_ = 1.36, *P* = 0.27; SS: *t*_3_ = 1.57, *P* = 0.22; comprehension: *t*_3_ = 0.79, *P* = 0.49; repetition: *t*_3_ = 0.09, *P* = 0.93; naming: *t*_3_ = 2.58, *P* = 0.08; BNT: *t*_3_ = 0.53, *P* = 0.63) (Fig. 4).

Preoperatively, the global network properties of the language and CO-FP networks did not differ between those well recovered patients and HCs. When compared with preoperative baseline values, severe disruptions within the language and CO-FP networks at the acute phase were observed (FC strength: language, *t*_16_ = 7.5, *P* < 0.001, CO-FP, *t*_16_ = 5.29, *P* < 0.001, paired *t*-test; gE: language, *t*_16_ = 8.11, *P* < 0.001, CO-FP, *t*_16_ = 6.89, *P* < 0.001; lE: language, *t*_16_ = , *P* < 0.001, paired *t*-test; CO-FP, *t*_16_ = 6.76, *P* < 0.001). As expected, the network properties for the language network and CO-FP network gradually increased from 1 month (FC strength: language, *t*_14_ = 3.74, *P* = 0.002, CO-FP, *t*_14_ = 2.98, *P* = 0.01; gE: *t*_14_ = 3.56, *P* = 0.003, CO-FP, *t*_14_ = 3.26, *P* = 0.006; lE: *t_14_* = 4.06, *P* = 0.001, CO-FP, *t*_14_ = 2.24, *P* = 0.04) to 3 months (FC strength: language, *t*_24_ = 3.3, *P* = 0.003, CO-FP, *t*_24_ = 1.81, *P* = 0.08; gE: *t*_24_ = 2.92, *P* = 0.008, CO-FP, *t*_24_ = 1.82, *P* = 0.08; lE: language, *t*_24_ = 3, *P* = 0.006, CO-FP, *t*_24_ = 1.09, *P* = 0.29) and 6 months (FC strength: language, *t*_3_ = 0.43, *P* = 0.7, CO-FP, *t*_3_ = 0.68, *P* = 0.55; gE: *t*_3_ = 0.78, *P* = 0.49, CO-FP, *t*_3_ = 1.1, *P* = 0.35; lE: language, *t*_3_ = 0.36, *P* = 0.74, CO-FP, *t_3_* = 0.81, *P* = 0.48).

When analysed by tumour grade, preoperative language scores were significantly lower in patients with both LGGs and HGGs than in control subjects (*Ps* < 0.05, Supplementary Fig. 2), while more severe language deficits were observed in HGGs. Also, more severe preoperative network disruptions were observed in HGGs. Patients with LGGs showed higher AQ ratios than patients with HGGs (two-sample *t*-test, *t*_32_ = 2.24, *P* = 0.03), but there was no significant difference in the amounts of AQ recovery after surgery. Postoperatively, the two subgroups showed very similar recovery patterns for language functions, although patients with LGGs showed sustained improvements 3–6 months after surgery. Similar recovery trajectories were observed for network properties in patients with both LGGs and HGGs. There was no significant difference in network recovery ratio or amount of network recovery after surgery between LGGs and HGGs.

### Patients with poor postoperative language recovery exhibited poor network normalization

For the six patients who did not recover well, three patients had severe preoperative language deficits (AQs < 85, range 67–85). All patients suffered severe language deficits in the acute phase (AQs < 65, range 50–65), and did not recover by 3 or 6 months (AQs < 81, Table 2 and Supplementary Fig. 3). Severe network disruptions were observed before and after surgery, and networks did not return to preoperative levels within 6 months.

### The correlation between AQ recovery ratio and network recovery ratio

No significant correlation was observed between age, education or WHO tumour grade with AQ recovery ratio or the amount of AQ recovery after surgery (partial *Rs* < 0.23, *Ps* > 0.21). No significant correlation was observed between the AQ recovery ratio and language network recovery ratio (partial *Rs* < 0.1, *P* > 0.58) or CO-FP network recovery ratio (partial *Rs* < 0.19, *Ps* > 0.32). No significant correlation was observed between the amounts of AQ recovery after surgery and amounts of language network recovery after surgery (partial *Rs* < 0.22, *Ps* > 0.25) or amounts of CO-FP network recovery after surgery (partial *Rs* < 0.2, *Ps* > 0.28).

### Location-dependent recovery of ipsihemispheric and interhemispheric connections

The recovery of network properties was mainly attributed to the recovery of interhemispheric and ipsihemispheric connections with fair and moderate functional connectivity values (Supplementary Figs 4 and 5).

**Figure 5:**
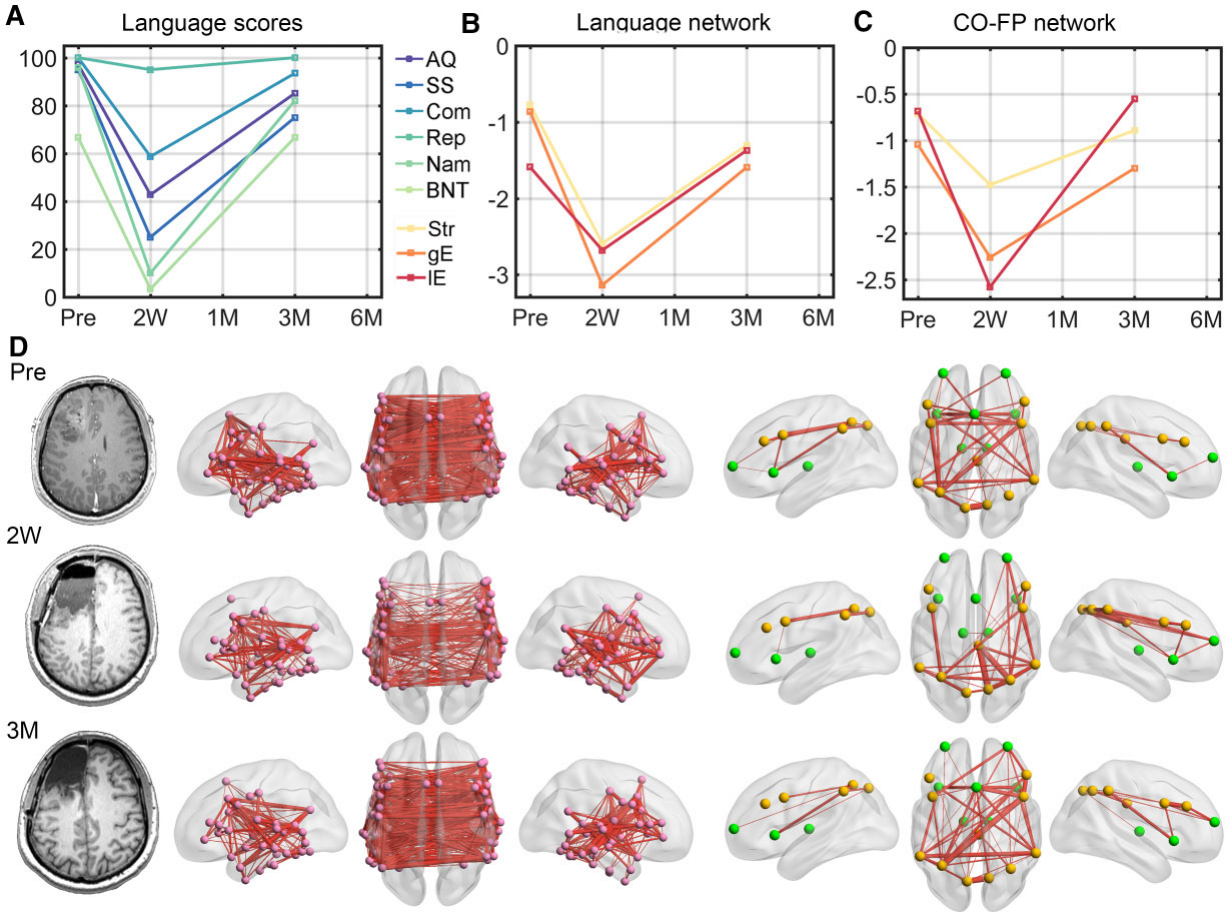
**A representative patient (male, 38 years) with frontal glioma (WHO III) who exhibited good language recovery and network normalization at 3 months after surgery.** (**A**) The language scores in each observation. For illustration purposes, the language scores of SS, Com, and BNT were scaled to 100. (**B**) and (**C**) The network properties for language and CO-FP networks. The values of each metric were Z-scored against the corresponding values in the control group. (**D**) The functional connectivity map in each observation. Left: language network; right: CO-FP network. Marked decreases of language functions, ipsihemispheric and interhemispheric functional connectivity, and global properties of both language and CO-FP networks were observed in the subacute phase. In the chronic phase, improvement of language functions paralleled proportional increases in global network properties. By 3 months after surgery, the two networks qualitatively resembled the preoperative networks. AQ, aphasia quotient; SS, spontaneous speech; Com, comprehension; BNT, Boston Naming Test; Str, strength; gE, global efficiency; lE, local efficiency.

The language and network recoveries of a representative patient (Patient 017, WHO III, frontal glioma) are shown in Fig. 5. The transient severe language deficits and network disruptions that were observed in the acute phase were well recovered by 3 months. Another two representative patients (Patient 096, WHO II, parietal glioma; Patient 051, WHO IV, temporal glioma) with different extents of network disruptions but similar recovery patterns are shown in Supplementary Figs 6 and 7. For these three patients, the disrupted and recovered connections were mainly around the tumour territory and the corresponding contralateral region, with fair and moderate functional connectivity values (Supplementary Fig. 8).

### The recovery of task activations of the language network

Patients 051 and 096 were also asked to perform a picture-naming task. Preoperatively, bilateral activations (*P* < 0.001, cluster size = 20, uncorrected, Supplementary Fig. 9) were observed in Patient 051, including frontal and parietal language areas and somatosensory areas. While, in the subacute phase, no activation was observed in the language network, and at 3 months after surgery, the bilateral activations in frontal and parietal language areas and somatosensory areas were well recovered. Preoperatively, activations in the left frontal cortex and subcortical regions were observed in Patient 096. While, in the acute phase, no activation was observed, and at 3 months after surgery, activations in the left frontal cortex, subcortical regions and somatosensory areas were observed.

### Results of the validation analysis

#### Network specificity

For those patients with language recovery, there was no significant preoperative network disruption within the DMN (Supplementary Fig. 10). Compared with the preoperative values, significant network disruptions of the DMN were observed in 2 weeks after surgery (FC strength: *t_16_* = 2.78, *P* = 0.01; gE: *t*_16_ = 2.8, *P* = 0.01; lE: *t*_16_ = 2.3, *P* = 0.04), which were partially recovered at 1 month (FC strength: *t*_14_ = 1.2, *P* = 0.25; gE: *t*_14_ = 0.23, *P* = 0.82; lE: *t*_14_ = 0.52, *P* = 0.61) and substantially recovered at 3 months (FC strength: *t*_24_ = 0.51, *P* = 0.61; gE: *t*_24_ = 0.24, *P* = 0.81; lE: *t*_24_ = −0.5, *P* = 0.62). At 6 months, significant network disruptions of the DMN were observed (FC strength: *t*_3_ = 3.11, *P* = 0.05; gE: *t*_3_ = 2.1, *P* = 0.13; lE: *t*_3_ = 5.76, *P* = 0.01) after surgery. Compared with the preoperative values, significant network disruptions of the MEN were observed in 2 weeks (FC strength: *t*_16_ = 3.83, *P* = 0.002; gE: *t*_16_ = 3.29, *P* = 0.005; lE: *t*_16_ = 3, *P* = 0.009) and 6 months after surgery (FC strength: *t*_3_ = 2.15, *P* = 0.12; gE: *t*_3_ = 2.06, *P* = 0.13; lE: *t*_3_ = 3.51, *P* = 0.04).

#### The effect of global signals

Without removing the global signals, differences in the networks after surgery, especially in the language network were observed, but the changing patterns did not synchronize with the changes in language scores (Supplementary Fig. 1^1^).

#### The effect of correlation threshold

Consistent with the main results, network normalizations were observed for both language and CO-FP networks under different correlation thresholds (Supplementary Fig. 1^2^).

## Discussion

In this study, we longitudinally investigated changes in functional networks during postoperative language recovery in patients with left cerebral gliomas. After awake surgery, transient declines of language functions and global network properties in the acute phase were observed in all 34 patients. Among patients who had severe language deficits after surgery, 28 out of 34 recovered to 80% of his or her baseline scores within 3 months or 85% within 6 months. This was coupled with increases in global network properties for the language and CO-FP networks. The network recoveries were tumour location dependent and were mainly attributed to the recovery of ipsihemispheric and interhemispheric connections with fair and moderate functional connectivity values. Six patients did not recover their language function well and all had severe network disruptions in the acute phase that tended to persist into the chronic phase.

Increased or restored functional connectivity or global network properties have been observed during cognitive recovery in other aetiologies, which is termed ‘network normalization’ hypothesis.^5,69,71–74^ A key finding of the present study is that for the two subgroups of patients with different language deficits and recovery ratios, synchronized network changes were observed. For the first time, our work provides empirical evidence to support the network normalization hypothesis for patients with glioma. We further showed that the network normalization phenomenon was network specific. Postoperative language recovery only synchronized the network normalization of language and control network, but not the DMN and MEN networks. Thus, although tumour resection can induce widespread network disruption,^75,76^ postoperative cognitive recovery was network specific.

We then showed that the transient declines and recoveries of network integrity mainly resulted from changes in the ipsihemispheric and interhemispheric connections. The loss of these neuronal signals has been a key feature of acute and progressive lesions that underlies cognitive deficits.^19,20,77–83^ Moreover, we also found that the disruptions and recovery of these connections were tumour location dependent. These findings are consistent with the network results of patients with glioma involving sensorimotor areas. Otten *et al.*^81^ showed that for a patient with SMA glioma who returned to full motor strength after 5 months, the average connectivity within the motor network exceeded preoperative connectivity, and the healing process involved ipsihemispheric and interhemispheric connections. Vassal *et al.*^83^ found that after surgery, the recovery of SMA syndrome in patients with glioma correlated with increases in interhemispheric connectivity within the sensorimotor network.

Regarding the tumour grade effect, more severe preoperative language deficits and network disruptions were observed in patients with HGGs than in those with LGGs. These findings are consistent with our previous works,^2,84–86^ which reflects the tumour grade-related neuroplasticity. Postoperatively, patients with either LGGs or HGGs showed very similar recovery patterns. Although the AQ recovery ratio of patients with LGGs was significantly higher than those with HGGs, no tumour grade-related recovery effect was observed. Due to the limited sample size, the difference in postoperative network recovery between patients with LGGs and HGGs remained to be elucidated in future study.^87^

We believe that the network plasticity revealed in this study holds important clinical significance. First, our results advance our understanding of brain plasticity and may explain why patients with gliomas that infiltrate eloquent areas can have their tumours resected without inducing permanent deficits.^13,15,25,88^ Second, our findings suggest that network integrity and ipsi- and interhemispheric functional connectivity may be markers for future language recovery, and thus network-based tools have the potential to predict or inform personalized language outcomes. Finally, the network-specific distributed process for language recovery that we revealed suggests novel targets for rehabilitative strategies, e.g. a more global strategy for boosting neural circuits rather than a single target.^83,89^

This study had several limitations. First, the sample size was relatively small (*n* = 34). The limited sample size and the heterogeneous lesion locations constrained our investigation of location effects on language recovery and our ambition to construct long-term prediction modes. Second, while we focused on functional network reorganization, disruptions of language-related WM tracts and how this affects language recovery remain a topic for future studies. Third, after surgery, patients may have cognitive deficits that span more than one domain (e.g. executive function, memory and attention^5,27^). Although we found that the CO-FP network showed plasticity during language recovery, whether the language outcome was influenced by deficits in the other domains remains to be determined. Fourth, we only described the brain–behaviour associations within 6 months because there were just three or four follow-up time points for each patient. More frequent follow-ups, especially for those with chronic cognitive deficits, remained to be elucidated in future studies. Finally, our patients were enrolled between 2015 and 2018. The pathological categorizations of samples before 2016 were based on the 2007 WHO classification system, while the rest were based on the 2016 WHO classification system. Future studies that utilize the new 2021 WHO classification system are needed to characterize the behaviour changes and network reorganizations for each molecular genetic subtyping.

## Conclusions

Preoperative language deficits and the time course of spontaneous recovery vary significantly among patients. Nevertheless, we found that changes in network integrity and functional connectivity are demonstrable of postoperative language recovery. The postoperative synchronization of functional network normalization and spontaneous language recovery in patients with glioma motivate us to develop tools for predicting longitudinal outcomes and global rehabilitative strategies.

## Supplementary Material

fcac046_Supplementary_Data

## Abbreviations

ABC =: Aphasia Battery of Chinese
AQ =: aphasia quotient
BNT =: Boston Naming Test
Com =: comprehension
HGG =: high-grade glioma
KPS =: Karnofsky performance scale
LGG =: low-grade glioma
SS =: spontaneous speech
