# Correction to: Longitudinal assessment of network reorganizations and language recovery in postoperative patients with glioma

**DOI:** 10.1093/braincomms/fcac326

**Published:** 2022-12-15

This is a correction to: Binke Yuan, Nan Zhang, Fangyuan Gong, Xindi Wang, Jing Yan, Junfeng Lu, Jinsong Wu, Longitudinal assessment of network reorganizations and language recovery in postoperative patients with glioma, *Brain Communications*, Volume 4, Issue 2, 2022, fcac046, https://doi.org/10.1093/braincomms/fcac046

In the originally published version of this manuscript, there was an error in the Graphical abstract and Figure 1, whereby the dotted line connecting the point of 2-week/2W was omitted.

In addition, the formula AQ = (S_ss_ + S_com_/23+S_rep_/10+S_nam_/10)*2 was omitted from the end of the following sentence: The aphasia quotient (AQ) (range, 0–100) can be calculated from these items to reflect the global severity: .

These errors have been corrected.

