# Supplementary material for: Longitudinal assessment of network reorganizations and language recovery in postoperative patients with glioma
Source: Brain Commun. 2022 Apr 6;4(2):fcac046. doi: 10.1093/braincomms/fcac046 (PMC8994117; doi:10.1093/braincomms/fcac046)
Supplement: fcac046_Supplementary_Data [file fcac046_supplementary_data.zip › Supplementary_Material 1.pdf]

Longitudinal assessment of network reorganizations and language recovery in postoperative patients with glioma

Binke Yuan, Nan Zhang, Fangyuan Gong, Xindi Wang, Jing Yan, Junfeng Lu, Jinsong Wu

SUPPLEMENTARY FIGURES

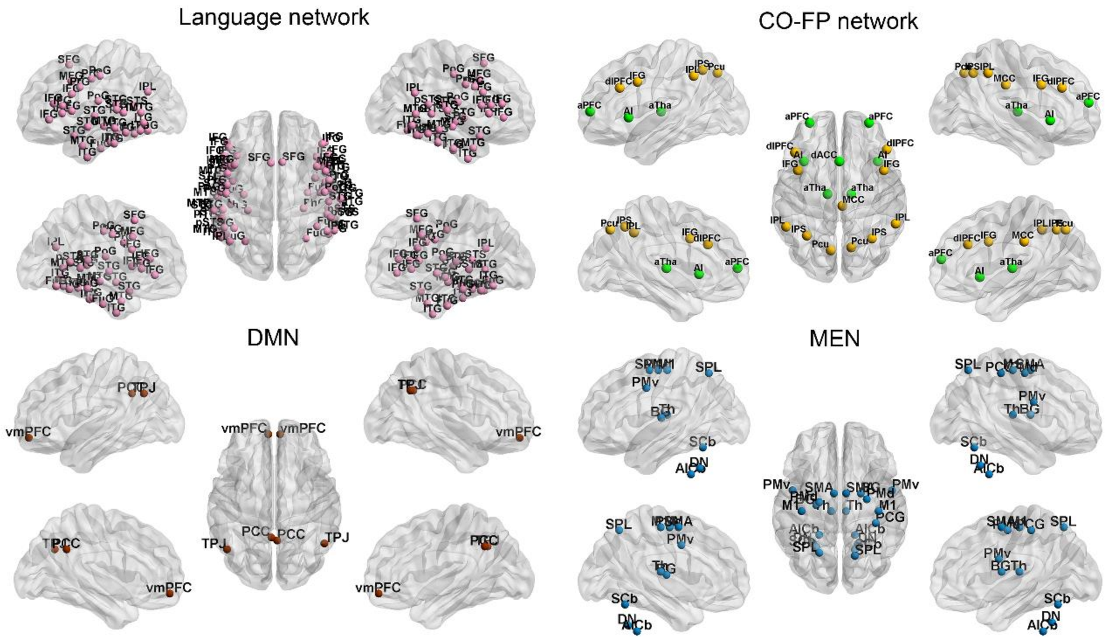

Supplementary Figure 1. Locations of networks of interest. Language network: Cortical parcels (68 nodes) involved in language function were extracted from the Brainnetome Atlas based on each parcel's behavioral domain and paradigm class metadata labels from the BrainMap database. Parcels of the left-lateralized language region homologs (i.e., parcels in the left frontal and temporal lobes) were also included. CO-FP network: Peaks of the 18 CO-FP ROIs were from Dosenbach et al. (2007). DMN: Peaks of the 6 DMN ROIs were from Anderson et al. (2011). MEN: Peaks of the 21 motor execution ROIs were from Wang et al. (2010). We defined ROIs based on peaks as 6-mm spheres centered on the peak coordinates.

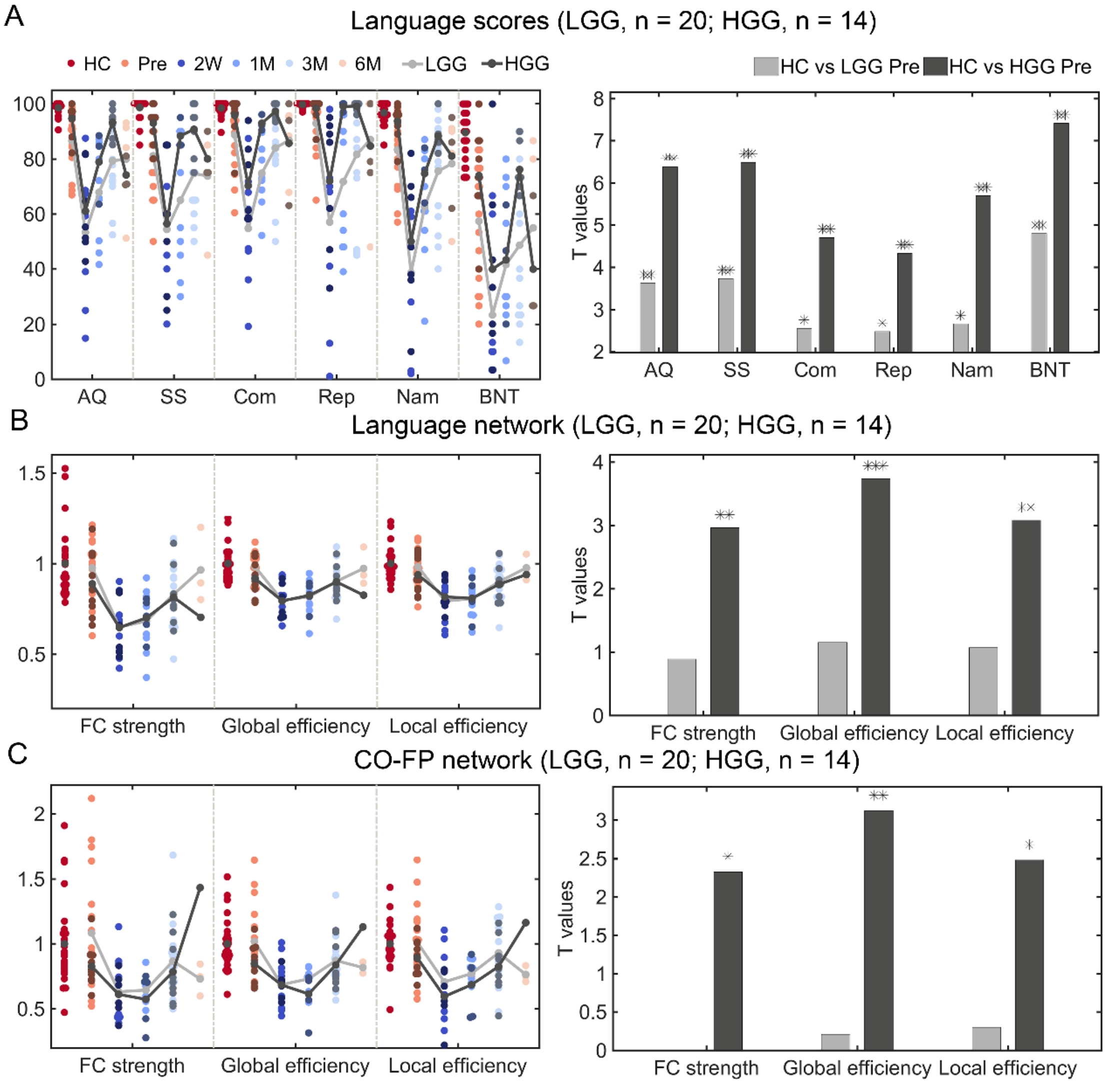

Supplementary Figure 2. Language scores and global network properties (Z-scores) for the 20 patients with LGGs and 14 patients with HGGs who showed good language recovery. In each panel, two solid lines (gray for LGGs and dark for HGGs) connecting the mean values of each measure over time were plotted. The *t* values were obtained by performing two-sample t-tests between HCs and patients with LGGs or HGGs. For illustration purposes, the language scores of SS, Com, and BNT were scaled. Note that the sample size vary in each observation, $n = 11$ with LGG, $n = 6$ with HGG in two 2weeks, $n = 10$ with LGG, $n = 5$ with HGG in 1-month, $n = 16$ with LGG, $n = 9$ with HGG in 3-month and $n = 3$ with LGG $n = 1$ with HGG in 6-month after surgery. Two-sample t-tests between HCs and preoperative patients with LGGs or HGGs were performed. *, $P < 0.05$; **, $P < 0.01$; ***, $P < 0.001$. AQ, aphasia quotient; SS: spontaneous speech; Com: comprehension; Rep: repetition; Nam, naming; BNT: Boston Naming Test; LGG, low-grade glioma; HGG, high-grade glioma.

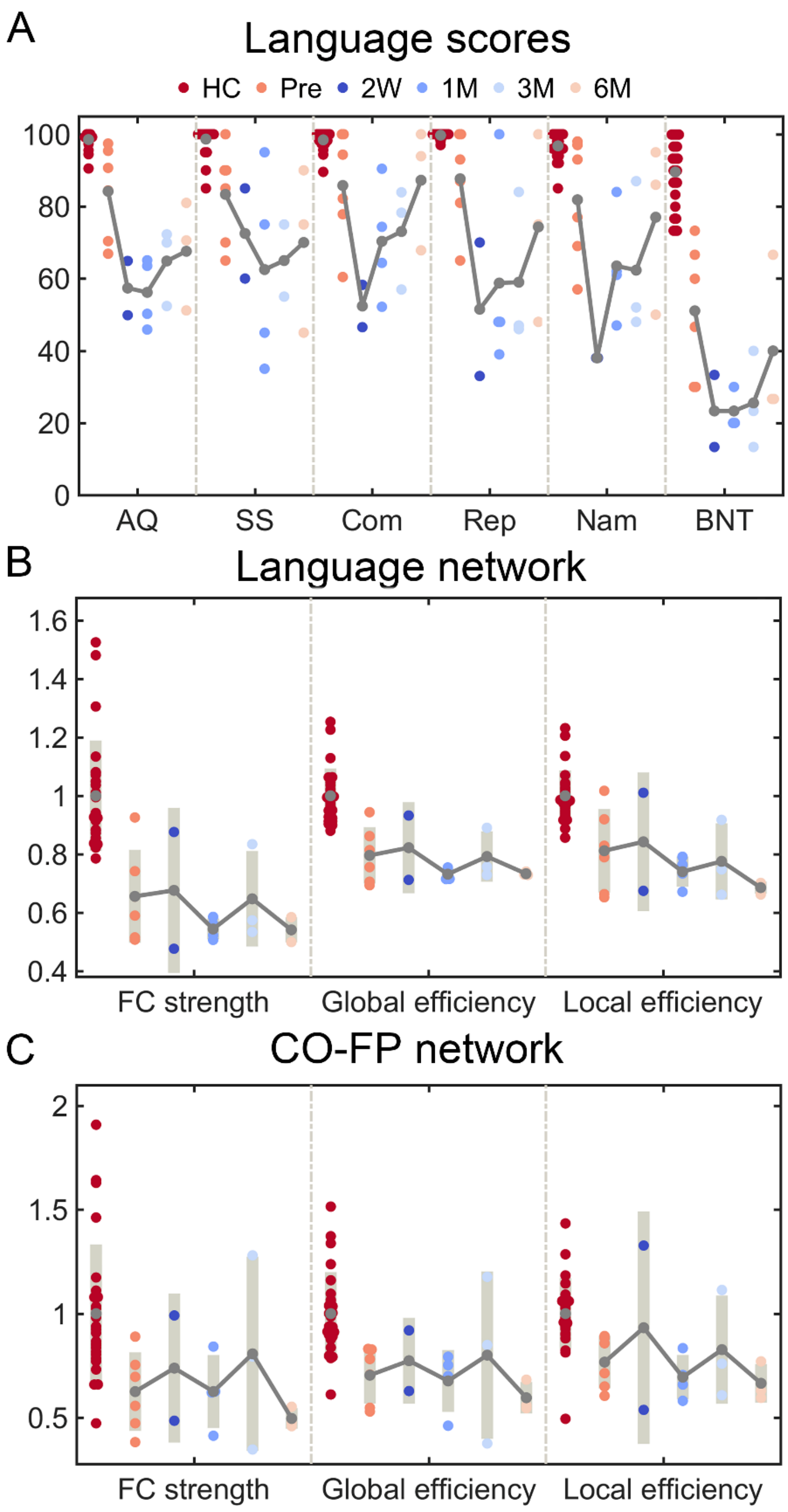

Supplementary Figure 3. Language scores and global network properties (Z-scores) for the 6 patients who showed poor language recovery.

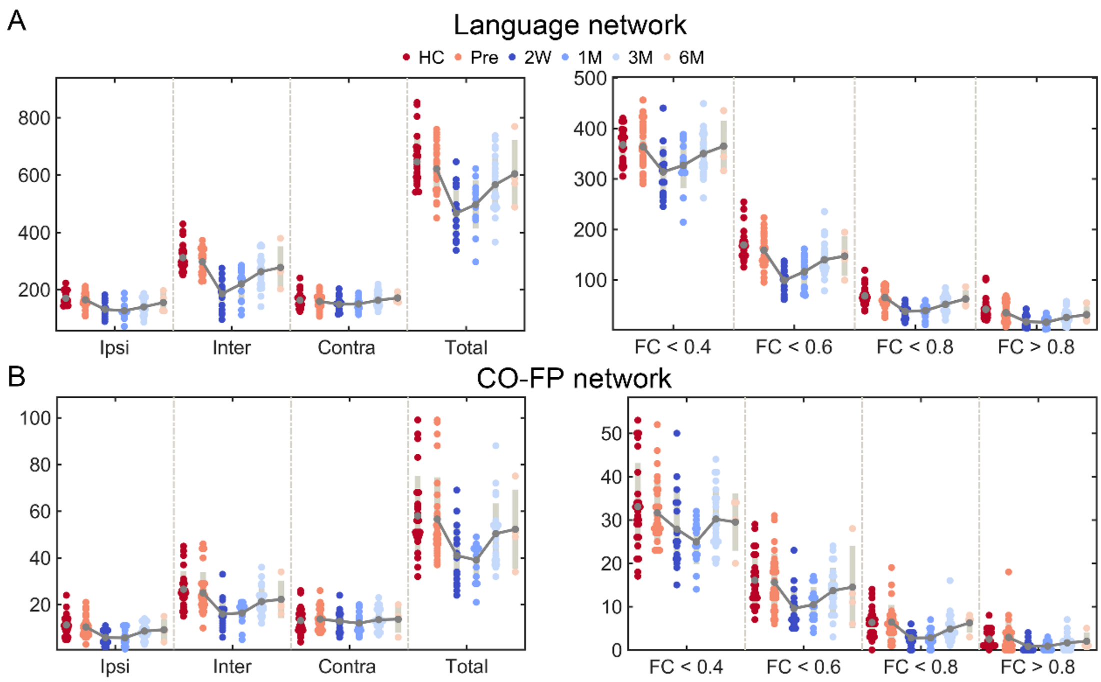

Supplementary Figure 4. The types of functional connectivity for the 28 patients with good language recovery. Ipsi: Ipsihemispheric; Inter: Interhemispheric; Contra: Contrahemispheric.

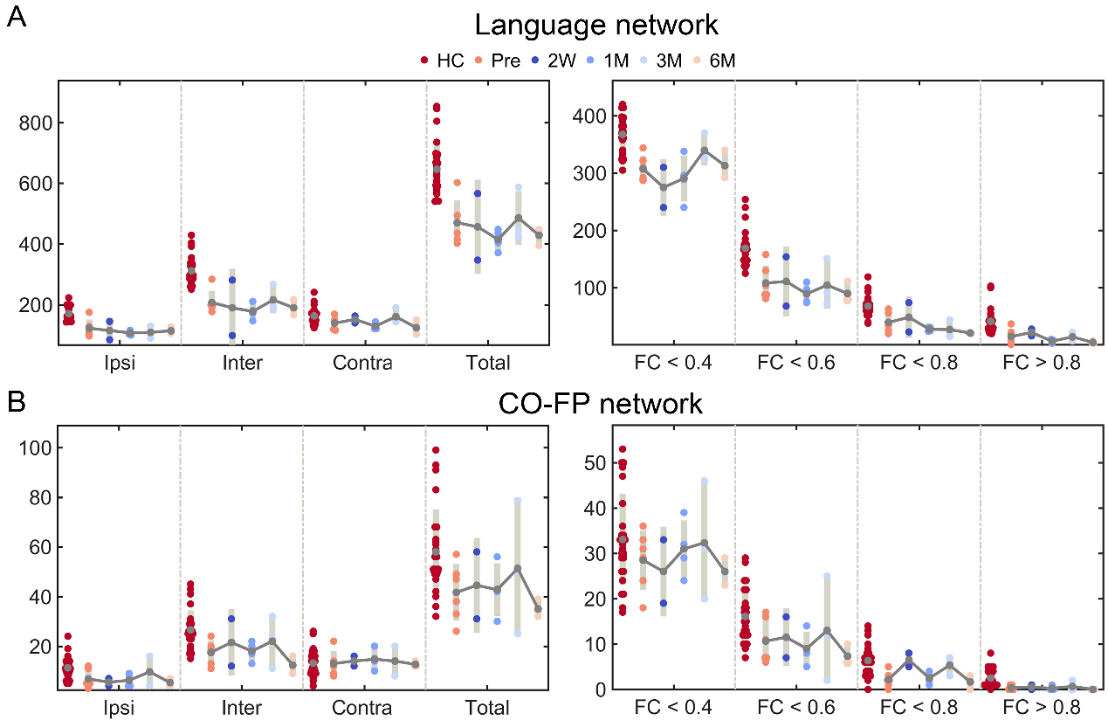

Supplementary Figure 5. The types of functional connectivity for the 6 patients with poor language recovery.

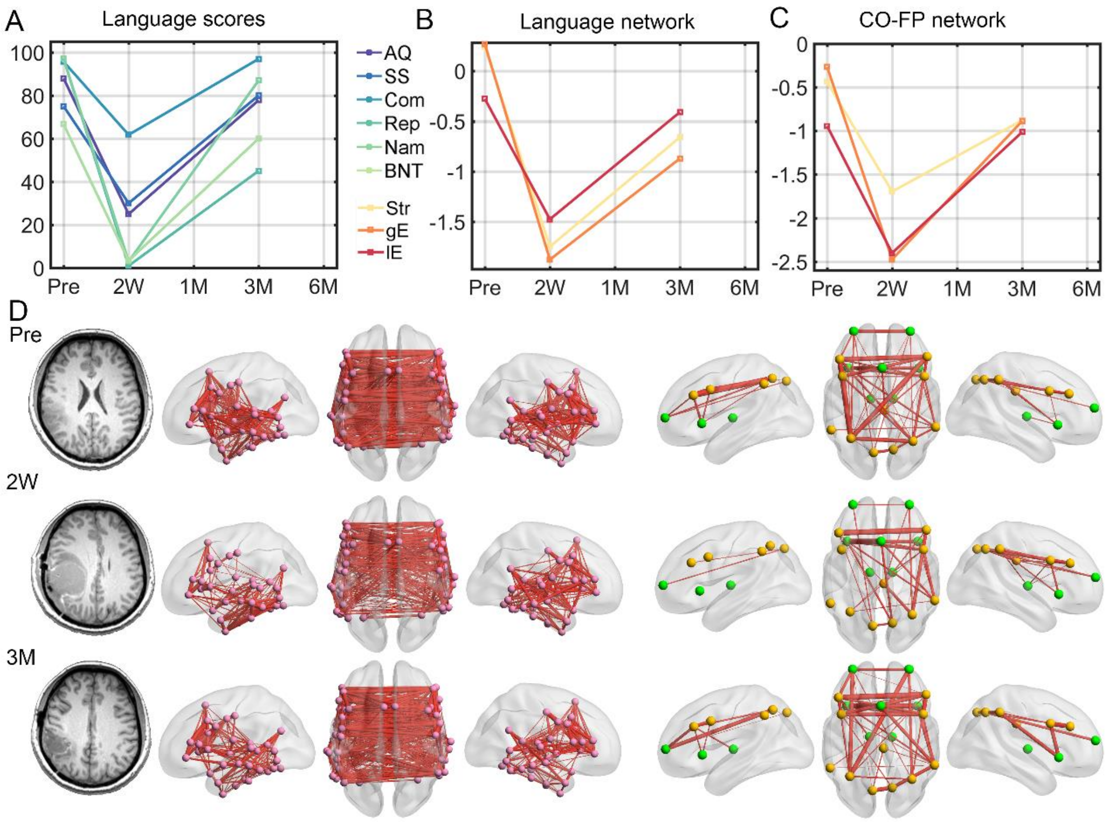

Supplementary Figure 6. A representative LGG patient (P096, female, 39 years) with parietal glioma (WHO II) showed good language recovery and network normalization in 3 months after surgery.

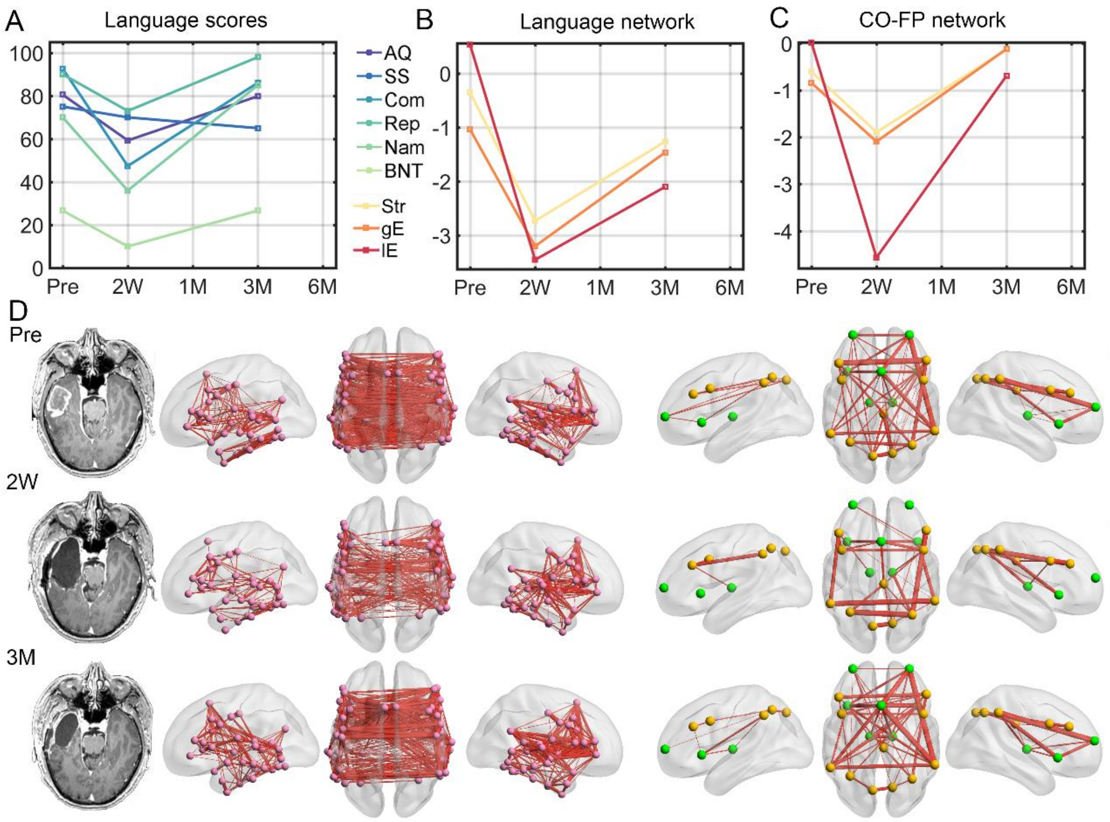

Supplementary Figure 7. A representative HGG patient (P051, male, 52 years) with temporal glioma (WHO WHO IV) showed good language recovery and network normalization in 3 months after surgery.

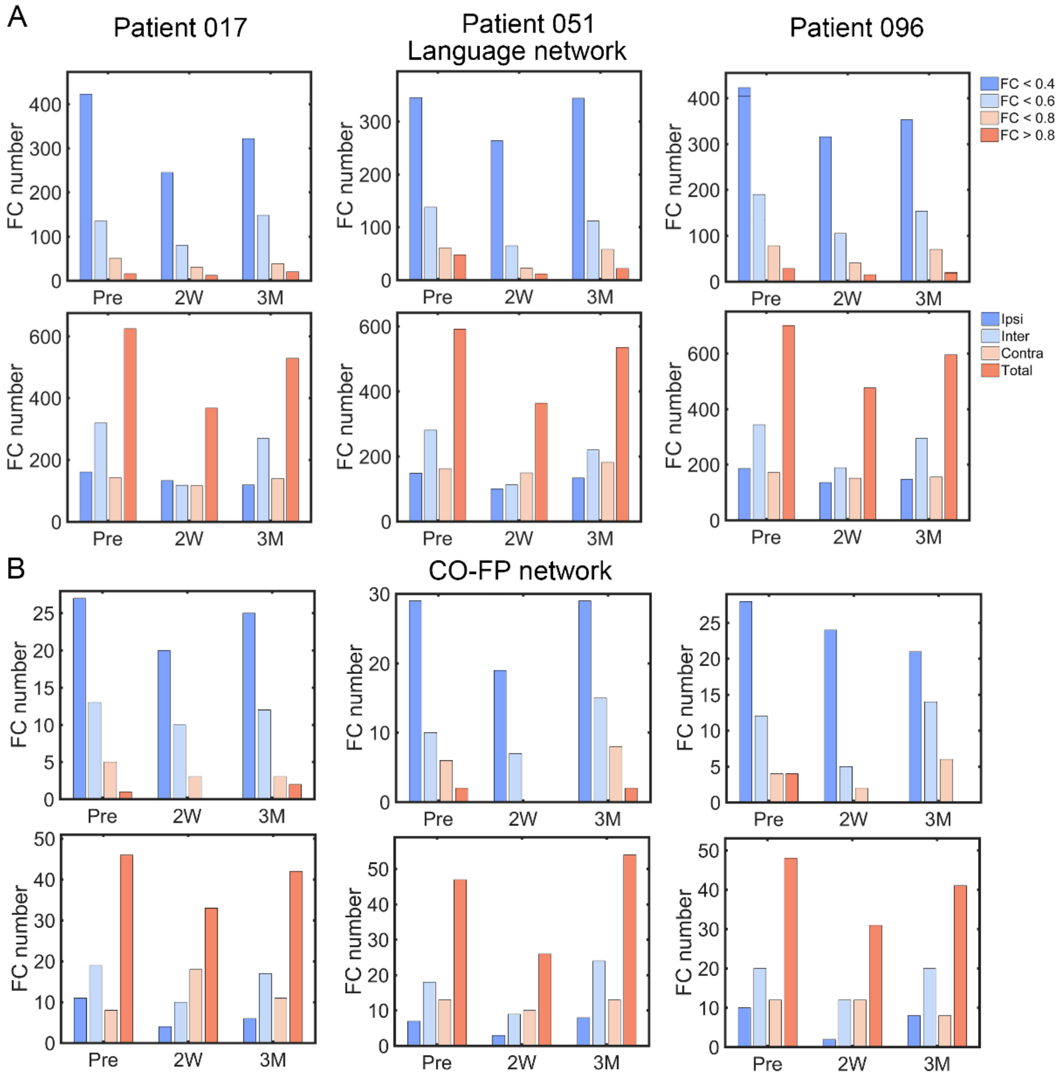

Supplementary Figure 8. The types of functional connectivity for patient 017, patient 051, and patient 096.

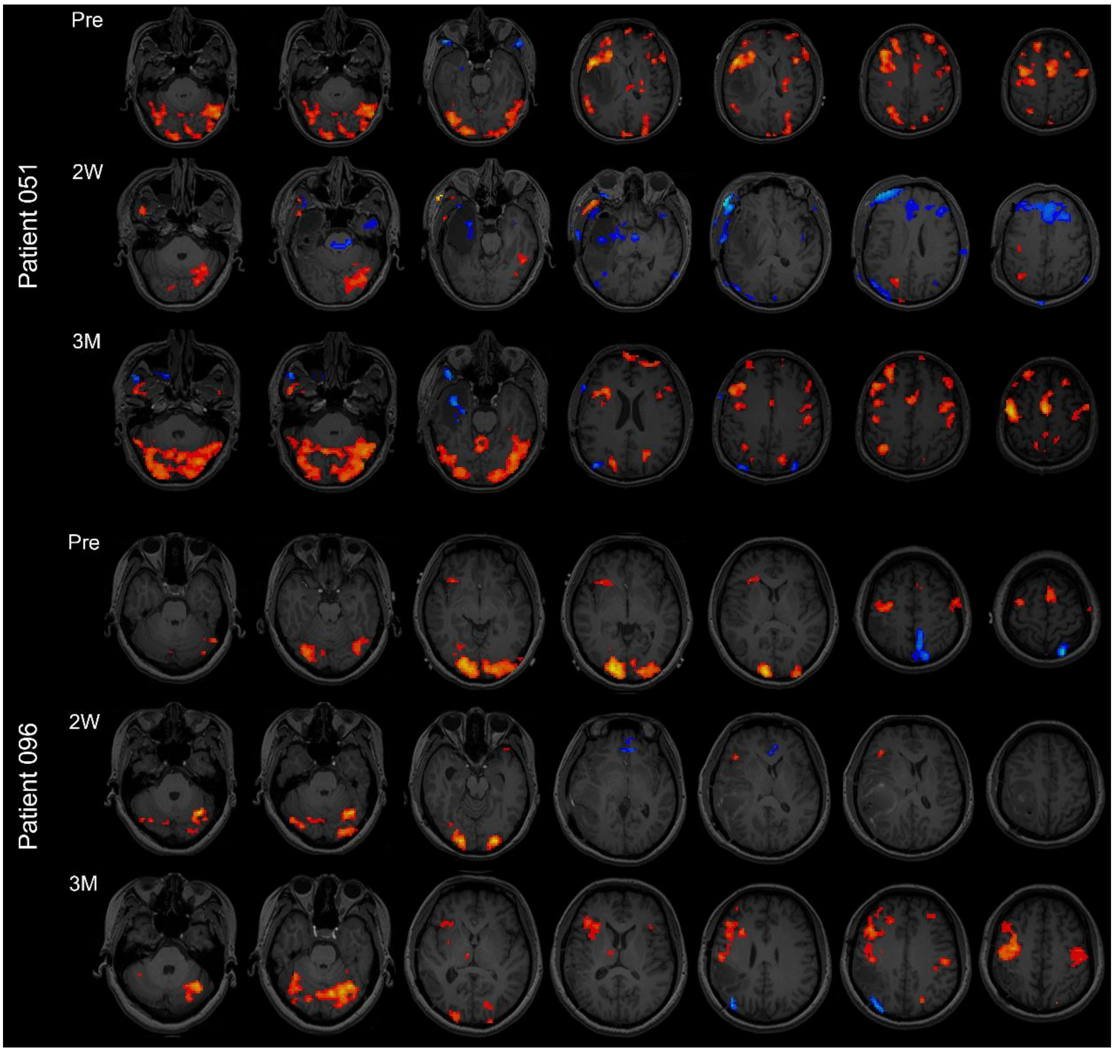

Supplementary Figure 9. The task activations of language network of a picture-naming task for patients 051 and 096. $P < 0.001$, cluster size = 20, uncorrected. The task was a blocked design, with alternating three task blocks and rest blocks ("rest – task – rest – task…"). Each block lasts 30 seconds with 10 trials. During the picture naming task, the pictures were projected onto a screen placed at the heads of the subjects through an MRI-compatible visual and auditory stimulation device. The subjects were required to "silently" name the pictures. After preprocessing (slice-timing, realign, smooth), a general linear model (GLM) with a task reference (i.e., boxcar time course) convoluted with the canonical hemodynamic response function was performed. The head motion parameters were also modeled in the GLM as nuisance regressors. The rest condition in each task was used as an implicit baseline in the model. Individual activation map (task > rest) was thresholded with an uncorrected P-value of 0.001 and a cluster extent of 20 voxels.

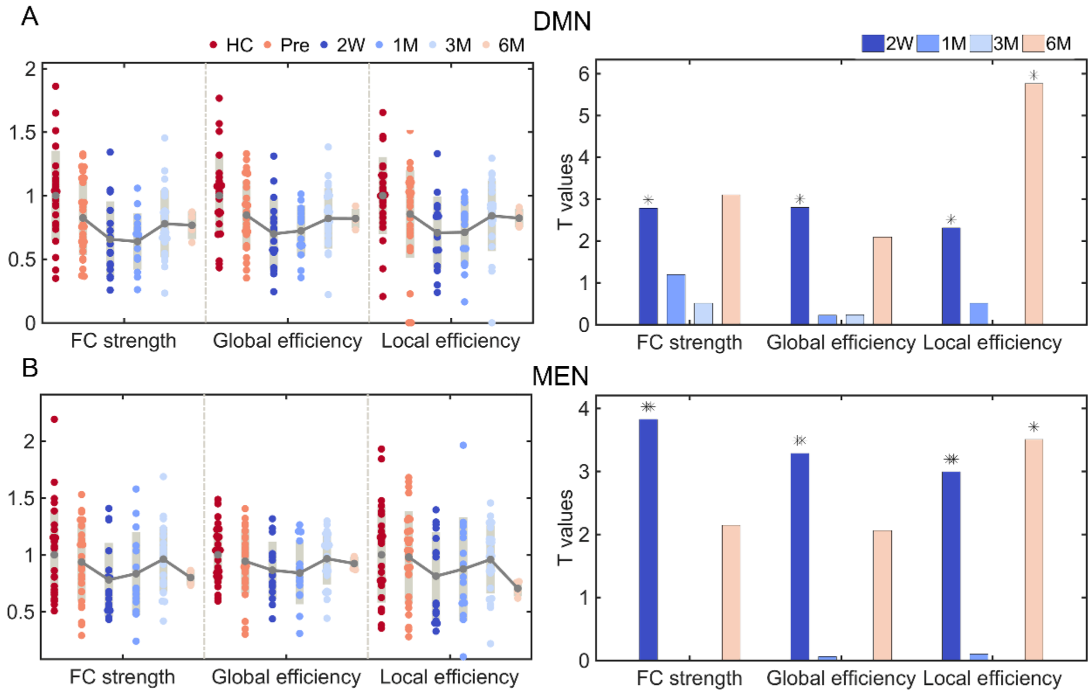

Supplementary Figure 10. The changes of global network properties of DMN and MEN for the 28 patients with good language recovery. Paired t-tests were performed. *, $P < 0.05$; **, $P < 0.01$; ***, $P < 0.001$.

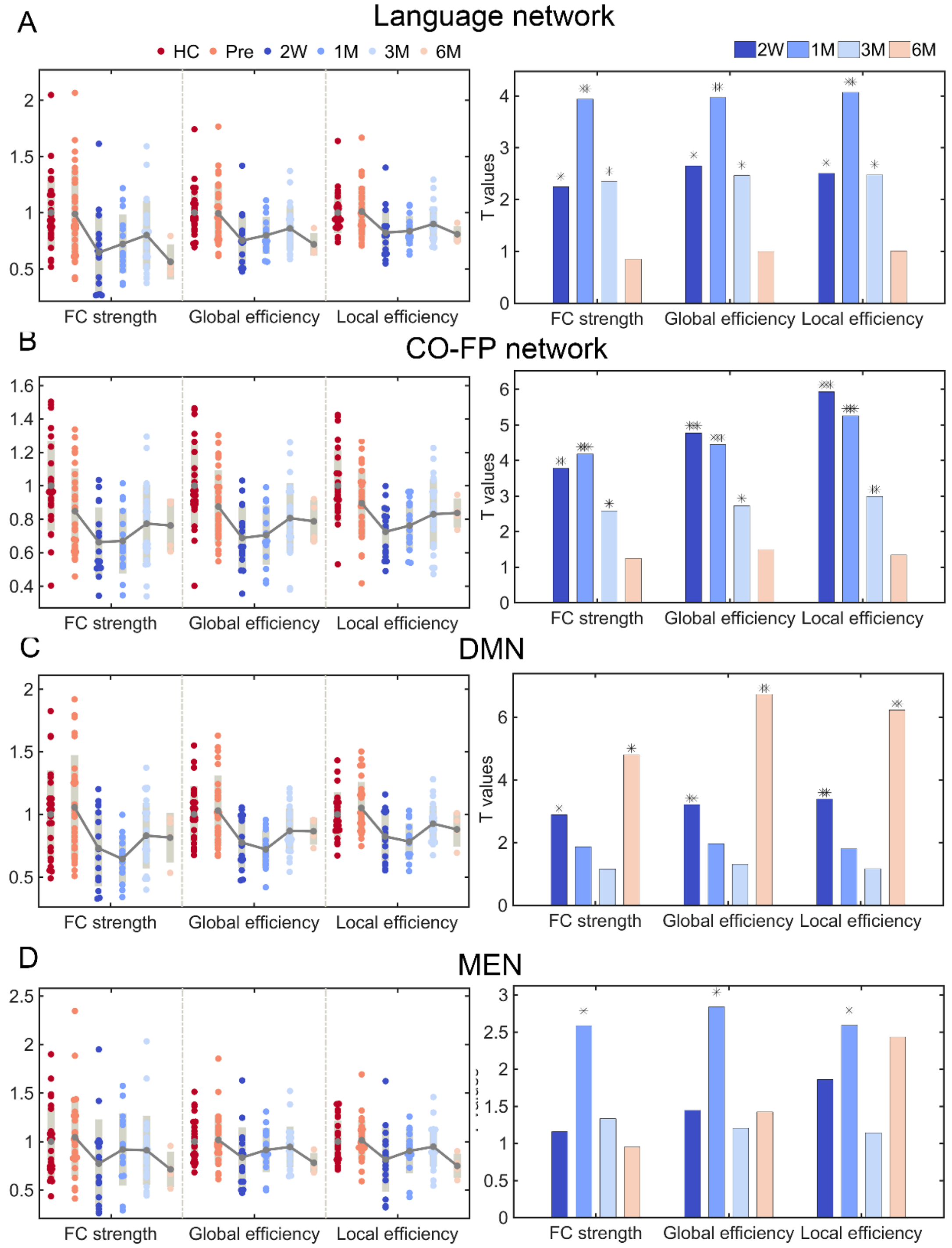

Supplementary Figure 11. The changes of global network properties for the 28 patients with good language recovery when did not perform global signal regression. Paired t-tests were performed. *, $P < 0.05$; **, $P < 0.01$; ***, $P < 0.001$.

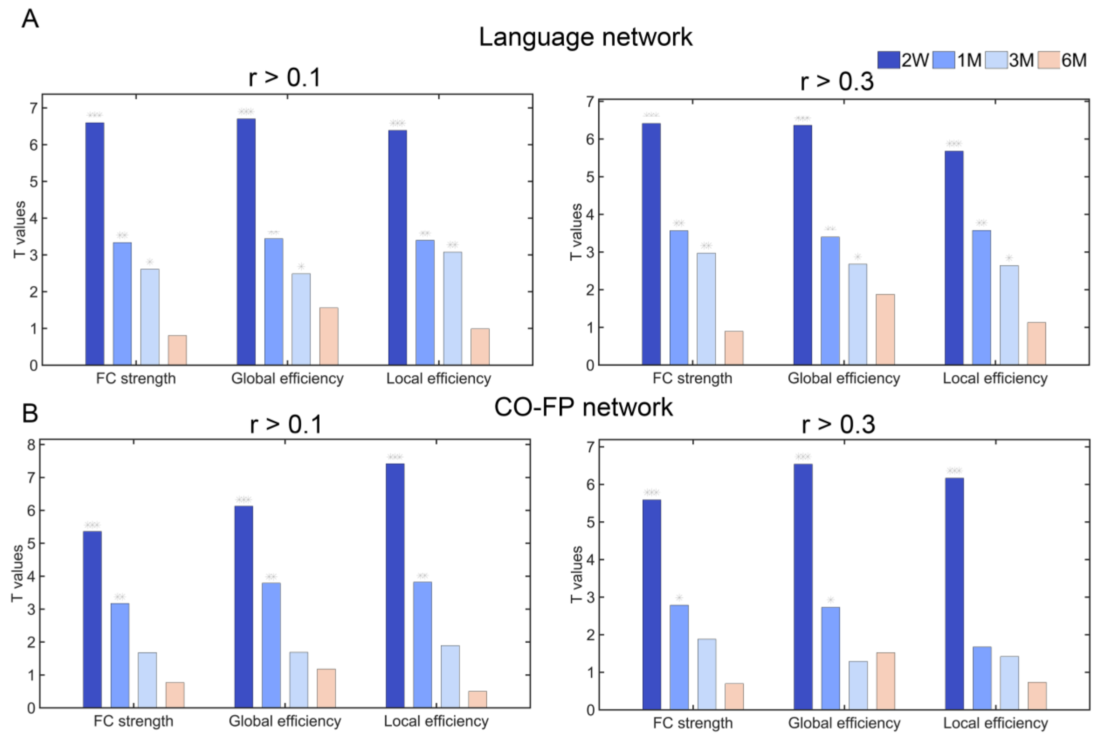

Supplementary Figure 12. Global network properties changes under different correlation thresholds. Paired t-tests were performed to track the longitudinal network normalization for the 28 patients with good language recovery. *, P < 0.05; **, P < 0.01; ***, P < 0.001.

SUPPLEMENTARY TABLES

Supplementary Table 1 Anatomical regions, language-related behavioral domains, and paradigm classes of the language network. Coordinates are in the standard Montreal Neurologic Institute Space.

ID	x	y	z	anatomy	behavioral domains	paradigm classes
1	-4.6	15.7	53.2	SFG	Execution. Speech, Phonology, Semantics, Speech	Word Generation (Covert and Overt)
2	7.0	16.4	54.7	SFG	Homologs of node 1	-
17	-41.9	13.6	36.4	MFG	Phonology, Semantics	Semantic. Monitor/Discrimination, Word Generation (Covert)
18	42.2	11.9	38.4	MFG	Homologs of node 17	-
29	-45.6	13.0	23.4	IFG	Phonology, Semantics, Speech, and Syntax	Phonological. Discrimination, Semantic. Monitor/Discrimination
30	45.0	15.7	25.0	IFG	Homologs of node 29	-
31	-47.6	31.6	13.6	IFG	Phonology, Semantics, Speech, and Syntax	Phonological. Discrimination, Semantic. Monitor/Discrimination, Word Generation (Covert and Overt)
32	47.8	35.1	13.2	IFG	Homologs of node 31	-
33	-52.2	22.5	11.4	IFG	Semantics, Speech, and Syntax	Reading (Covert), Semantic. Monitor/Discrimination, Word Generation (Covert and Overt)
34	54.2	23.9	11.8	IFG	Homologs of node 33	-
35	-49.1	36.3	-2.8	IFG	Semantics, Speech, and Syntax	Semantic. Monitor/Discrimination, Word Generation (Covert)
36	50.8	36.8	-0.7	IFG	Homologs of node 35	-
37	-39.5	22.9	3.7	IFG	Phonology, Semantics, Speech, and Syntax	Semantic. Monitor/Discrimination, Word Generation (Covert)
38	42.1	22.0	3.2	IFG	Homologs of node 37	-
39	-51.3	13.2	6.0	IFG	Phonology, Semantics, Speech	Music. Comprehension/Production, Recitation/Repetition. (Covert), Word Generation (Covert)
40	53.6	14.3	11.8	IFG	Homologs of node 39	-
53	-49.5	-7.1	38.8	PrG	Execution. Speech	Reading (Overt), Recitation/Repetition. (Overt)
54	54.9	-2.0	33.3	PrG	Execution. Speech	Reading (Overt), Recitation/Repetition. (Overt)
63	-49.1	4.7	30.5	PrG	Orthography, Phonology, Semantics, Speech and Syntax	Phonological. Discrimination, Reading (Covert)
64	51.1	7.2	30.9	PrG	Homologs of node 63	-
71	-53.7	-32.1	12.4	STG	Execution. Speech, Phonology, and Speech	Music. Comprehension/Production, Passive Listening, Phonological.

						Discrimination, Reading (Overt), Recitation/Repetition. (Covert and Overt)
72	54.5	-23.7	10.6	STG	Execution. Speech, Phonology	Music. Comprehension/Production, Passive Listening, Phonological. Discrimination, Recitation/Repetition. (Overt)
73	-50.1	-10.3	1.1	STG	Execution. Speech, Phonology, Speech	Music. Comprehension/Production, Passive Listening, Phonological. Discrimination, Reading (Overt), Recitation/Repetition. (Overt)
74	51.1	-3.7	-0.9	STG	Execution. Speech,	Music. Comprehension/Production, Passive Listening, Recitation/Repetition. (Overt)
75	-62.8	-32.8	7.4	STG	Execution. Speech, Phonology, Semantics, Speech	Passive Listening, Phonological. Discrimination, Reading (Overt), Semantic. Monitor/Discrimination
76	66.5	-20.8	6.6	STG	Execution. Speech, Phonology, Speech	Music. Comprehension/Production, Passive Listening, Phonological. Discrimination, Reading (Overt)
77	-45.1	10.5	-19.4	STG	Homologs of node 78	-
78	47.1	12.3	-19.7	STG	Speech	Film Viewing, Passive Listening
79	-55.1	-3.2	-10.1	STG	Execution. Speech, Phonology, Semantics, Speech	Music. Comprehension/Production, Passive Listening, Phonological. Discrimination, Reading (Overt)
80	55.8	-12.5	-5.2	STG	Execution. Speech, Phonology, Semantics, Speech	Music. Comprehension/Production, Passive Listening, Phonological. Discrimination, Reading (Overt), Semantic. Monitor/Discrimination
81	-65.2	-30.9	-11.3	MTG	Semantics	Semantic. Monitor/Discrimination
82	64.5	-29.2	-13.2	MTG	Homologs of node 81	-
83	-53.2	2.2	-29.6	MTG	Language	Semantic. Monitor/Discrimination, Reading (Covert)
84	51.1	5.7	-31.8	MTG	Language	Passive Listening
85	-58.9	-57.6	4.3	MTG	Semantics and Syntax	Semantic. Monitor/Discrimination, Word Generation (Overt)
86	60.1	-53.3	2.9	MTG	Homologues of node 86	Film Viewing
87	-58.5	-19.8	-9.4	MTG	Phonology, Semantics, Speech, and Syntax	Passive Listening, Phonological. Discrimination, Reading (Covert), Semantic. Monitor/Discrimination
88	58.3	-15.4	-10.1	MTG	Semantics and Speech	Passive Listening, Phonological. Discrimination
89	-45.5	-26.7	-26.1	ITG	Orthography and Semantics	Reading (Covert), Semantic. Monitor/Discrimination
90	45.8	-14.6	-32.4	ITG	Homologs of node 89	-

91	-50.5	-57.0	-14.1	ITG	Phonology and Semantics	Naming (Overt)
92	53.5	-52.4	-18.5	ITG	Phonology and Semantics	-
93	-43.7	-2.9	-41.4	ITG	Semantics	Semantic. Monitor/Discrimination
94	40.8	0.31	-42.8	ITG	Homologs of node 94	-
97	-55.2	-60.3	-6.0	ITG	Semantics and Speech	Film Viewing, Naming (Overt)
98	54.2	-56.9	-8.6	ITG	Homologs of node 97	-
99	-58.8	-42.1	-16.0	ITG	Orthography and Semantics	Naming (Overt), Reading (Covert), Semantic. Monitor/Discrimination, Word Generation (Overt)
100	60.5	-40.5	-17.1	ITG	Homologs of node 99	-
101	-54.9	-30.3	-27.4	ITG	Semantics	-
102	53.7	-30.3	-26.3	ITG	Homologs of node 101	-
103	-32.4	-16.6	-32.3	FuG	Semantics and Speech	Naming (Overt), Semantic. Monitor/Discrimination
104	33.1	-14.6	-34.1	FuG	Semantics	Naming (Overt), Semantic. Monitor/Discrimination
105	-30.6	-64.4	-14.1	FuG	Orthography, Semantics, Speech	Naming (Covert and Overt)
106	31.3	-61.4	-13.7	FuG	Language	Naming (Covert and Overt)
107	-42.3	-50.9	-17.3	FuG	Orthography, Phonology, Semantics, Speech	Naming (Covert and Overt), Phonological. Discrimination, Reading (Covert) and Semantic. Monitor/Discrimination
108	42.7	-49.1	-18.6	FuG	Orthography, Semantics	Naming (Covert)
113	-28.3	-32.6	-16.9	PhG	Semantics	Naming (Overt), Semantic. Monitor/Discrimination
114	28.8	-30.7	-17.5	PhG	Homologs of node 113	Passive Listening, Semantic. Monitor/Discrimination
121	-54.4	-39.8	4.2	pSTS	Phonology, Semantics, Speech, Syntax	Passive Listening, Phonological. Discrimination, Reading (Covert), Semantic. Monitor/Discrimination, Word Generation (Covert and Overt)
122	52.9	-36.8	3.1	pSTS	Execution. Speech, Phonology, Semantics, and Speech	Passive Listening, Phonological. Discrimination, Reading (Overt)
123	-52.4	-50.3	10.8	pSTS	Orthography, Semantics, Speech, and Syntax	Reading (Covert), Semantic. Monitor/Discrimination
124	56.5	-40.1	12.5	pSTS	Homologues of node 123	Passive Listening
143	-46.8	-64.7	25.8	IPL	Language	Semantic. Monitor/Discrimination,
144	53.0	-54.1	24.4	IPL	Homologs of node 143	-
155	-50.4	-15.8	42.1	PoG	Execution. Speech	Recitation/Repetition. (Overt)
156	50.3	-14.2	43.7	PoG	Execution. Speech	Reading (Overt), Recitation/Repetition. (Overt)
157	-55.8	-14.0	16.2	PoG	Execution. Speech	Recitation/Repetition. (Overt)
158	55.2	-10.2	15.0	PoG	Execution. Speech	Recitation/Repetition. (Overt)

SFG, superior frontal gyrus; *MFG*, middle frontal gyrus; *IFG*, inferior frontal gyrus; *PrG*, precentral gyrus; *STG*, superior temporal gyrus; *MTG*, middle temporal gyrus; *ITG*, inferior temporal gyrus; *FuG*, fusiform gyrus; *PhG*, hippocampal gyrus; *pSTS*, posterior superior temporal sulcus; *IPL*, inferior parietal lobule. *PoG*, postcentral gyrus. The ID is the number of the parcel in the Brainnetome atlas (Fan et al., 2016). Here we only summarized the language-related behavioral domains and paradigm classes; the full behavioral domains and paradigm classes for each node are available at http://atlas.brainnetome.org/bnatlas.php.

Supplementary Table 2 Anatomical regions of CO-FP network and coordinates in the standard Montreal Neurologic Institute Space

	x	y	z	anatomy
CO	-36	18	0	AI
	39	18	-3	AI
	0	18	45	dACC
	-30	57	6	aPFC
	30	57	15	aPFC
	-12	-15	6	aTha
	12	-15	6	aTha
FP	-33	-57	48	IPS
	33	-60	45	IPS
	-42	9	36	IFG
	45	9	33	IFG
	-9	-72	45	Pcu
	12	-69	45	Pcu
	-54	-48	42	IPL
	57	-45	45	IPL
	-45	27	30	dlPFC
	48	30	30	dlPFC
	3	-27	33	MCC

AI, anterior insula; *dACC*, dorsal anterior cingulate cortex; *aPFC*, anterior prefrontal cortex; *aTha*, anterior thalamus; *IPS*, intraparietal sulcus; *IFG*, inferior frontal gyrus; *Pcu*, precuneus; *IPL*, inferior parietal lobule; *dlPFC*, dorsal lateral prefrontal cortex; *MCC*, middle cingulate cortex.

Supplementary Table 3 Anatomical regions of DMN and coordinates in the standard Montreal Neurologic Institute Space

x	y	z	anatomy
-4	-52	32	PCC
-5	-55	-13	vmPFC
-49	-62	34	TPJ
4	-53	35	PCC
5	55	-13	vmPFC
50	-57	36	TPJ

PCC, posterior cingulate cortex; *vmPFC*, ventromedial prefrontal cortex; *TPJ*, temporoparietal junction.

Supplementary Table 4 Anatomical regions of motor execution network and coordinates in the standard Montreal Neurologic Institute Space

x	y	z	anatomy
16	-59	-21	SCb
-38	-22	56	M1
38	-22	56	M1
-10	-20	11	Th
-22	-62	54	SPL
-5	-4	57	SMA
5	-4	57	SMA
28	-10	54	PMd
-49	-1	38	PMv
-25	-56	-21	SCb
16	-66	57	SPL
19	-55	-39	DN
53	0	25	PMv
-22	-45	-49	AICb
16	-45	-49	AICb
37	-34	53	PCG
-22	-13	57	PMd
22	-2	12	BG
-25	-14	8	BG
7	-20	11	Th
-28	-55	-43	DN

SCb: superior cerebellum; *M1*: primary motor cortex; *Th*: thalamus; *SPL*: superior parietal lobule; *SMA*: supplementary motor area; *PMd*: dorsolateral premotor cortex; *PMv*: ventrolateral premotor cortex; *DN*: dentate nucleus; *AICb*: anterior inferior cerebellum; *PCG*: postcentral gyrus; *BG*: basal ganglia.
